# Piezoelectric Heterojunctions as Bacteria‐Killing Bone‐Regenerative Implants

**DOI:** 10.1002/adma.202413171

**Published:** 2024-10-25

**Authors:** Youzhun Fan, Jinxia Zhai, Zhengao Wang, Zhaoyi Yin, Haoyan Chen, Maofei Ran, Zurong Zhu, Yubin Ma, Chengyun Ning, Peng Yu, Chuanbin Mao

**Affiliations:** ^1^ School of Materials Science and Engineering Guang Dong Engineering Technology Research Center of Metallic Materials Surface Functionalization National Engineering Research Center for Tissue Restoration and Reconstruction Medical Devices Research and Testing Center South China University of Technology Guangzhou 510641 P. R. China; ^2^ Faculty of Materials Science and Engineering Kunming University of Science and Technology Kunming 650093 P. R. China; ^3^ Department of Biomedical Engineering The Chinese University of Hong Kong Sha Tin Hong Kong SAR P. R. China

**Keywords:** antibacterial therapy, implants, infected bone defects, piezoelectric heterojunctions, tissue regeneration

## Abstract

Heterojunctions are widely used in energy conversion, environmental remediation, and photodetection, but have not been fully explored in regenerative medicine. In particular, piezoelectric heterojunctions have never been examined in tissue regeneration. Here the development of piezoelectric heterojunctions is shown to promote bone regeneration while eradicating pathogenic bacteria through light‐cellular force‐electric coupling. Specifically, an array of heterojunctions (TiO_2_/Bi_2_WO_6_), made of piezoelectric nanocrystals (Bi_2_WO_6_) decorating TiO_2_ nanowires, is fabricated as a biocompatible implant. Upon exposure to near‐infrared light, the piezoelectric heterojunctions generate reactive oxygen species and heat to kill bacteria through photodynamic and photothermal therapy, respectively. Meanwhile, the mechanical forces of the stem cells grown on the implant trigger the heterojunctions to produce electric fields that further promote osteogenesis to achieve osteointegration. The heterojunctions effectively suppress postoperative recurrent infections while promoting osseointegration through the local electric fields induced by cells. Therefore, the piezoelectric heterojunctions represent a promising antibacterial tissue‐regenerative implant.

## Graphical abstract

TiO_2_/Bi_2_WO_6_ piezoelectric heterojunctions with engineered oxygen vacancies, interfacial built‐in electric fields and piezoelectric properties are constructed as bone implants. The heterojunctions achieve highly efficient and dynamic antimicrobial functions through near infrared (NIR)‐triggered photothermal and photodynamic therapies. Meanwhile, the mechanical forces of the stem cells grown on the implants trigger the heterojunctions to produce electric fields in situ, significantly promoting osteogenesis.

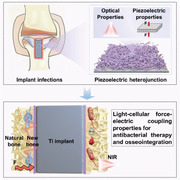

## 1 Introduction

Heterojunctions composed of two different semiconductors possess unique electronic properties at their interface, which can trigger diverse physical effects including heat, light, and electricity, often not found in “natural” materials.^[^ ^1^ ^]^ Heterojunctions are widely utilized in various fields, such as energy conversion,^[^ ^2^ ^]^ environmental remediation,^[^ ^3^ ^]^ and optoelectronic detection.^[^ ^4^ ^]^ Recently, the application of heterojunctions in the field of regenerative medicine has been drawing increasing attention.^[^ ^5^ ^]^ For example, Wang et al. constructed CoFe_2_O_4_@MnFe_2_O_4_ core–shell heterojunction magnetic nanoparticles to induce thermogenesis under a magnetic field.^[^ ^6^ ^]^ This promotes the expression of heat shock proteins and activates osteogenic pathways. To promote bone regeneration using the electrical effect of heterojunction, a TiO_2_/Bi_2_O_3_ nano‐heterojunction was constructed on a titanium bone implant to exhibit a built‐in electric field that regulates osteogenic differentiation.^[^ ^7^ ^]^ Additionally, porous/non‐porous heterojunctions are developed to stimulate the recovery of damaged neural tissues through light‐induced electric currents.^[^ ^8^ ^]^ These studies demonstrate the significant potential of heterojunction materials in regenerative medicine.

**Scheme 1:**
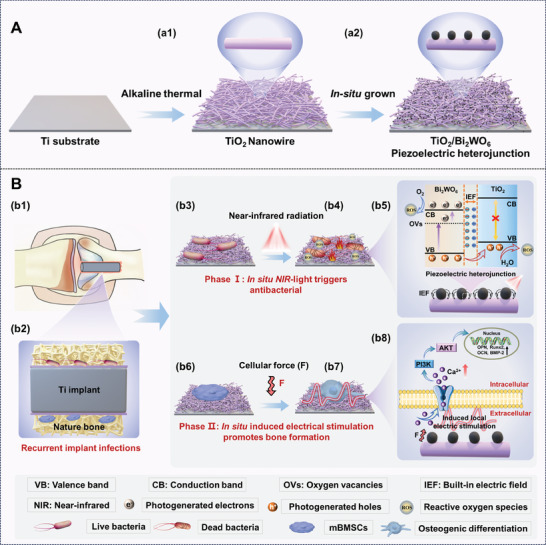
Schematic illustration of piezoelectric heterojunctions (TiO_2_/Bi_2_WO_6_) as implants for recurrent infection treatment and osteointegration. A) Schematic illustration of the in situ construction of TiO_2_/Bi_2_WO_6_ on the titanium implant surface. a1) Preparation of TiO_2_ nanowires on the titanium implant surface by alkaline thermal treatment. a2) In situ hydrothermal growth of Bi_2_WO_6_ nanocrystals on the TiO_2_ nanowires, generating TiO_2_/Bi_2_WO_6_ on the implant surface. B) NIR light‐triggered ROS (facilitated by the built‐in electric field, IEF) and heat production for photodynamic and photothermal antibacterial therapy, and cellular force‐induced electrical stimulation for bone formation. b1,b2) Implantation of TiO_2_/Bi_2_WO_6_ with bacterial infection in bone defect model. b3) Live bacteria grown on the surface of TiO_2_/Bi_2_WO_6_. b4) Bacterial death due to photothermal and photodynamic therapy. b5) Under NIR irradiation, oxygen vacancies (enabling the heterojunctions to absorb NIR light to produce heat for photothermal antibacterial therapy) are formed in Bi_2_WO_6_ by using ethylene glycol to remove lattice oxygen ions, and IEF induces the separation of electron‐hole pairs from Bi_2_WO_6_ to TiO_2_, which promotes the ROS production for photodynamic antibacterial therapy. b6) Growth of bone marrow mesenchymal stem cells (mBMSCs) on TiO_2_/Bi_2_WO_6_. b7) The electrical stimulation, generated by cellular force (F) due to the presence of piezoelectric Bi_2_WO_6_, facilitates osteogenic differentiation of mBMSCs and thus effectively promotes osseointegration. b8) The force from mBMSCs generates a local electric field that promotes inward Ca^2+^ flux, which activates the PI3K‐AKT signaling pathway, upregulates the expression of osteogenesis‐related genes (OCN, OPN, Runx2, and BMP‐2), and facilitates osteogenic differentiation.

However, the piezoelectric heterojunction for regenerative medicine remains rarely reported, in sharp contrast to the fact that many important tissues in the human body, such as bones, tendons, ligaments, and skin, are formed by the assembly of piezoelectric collagen fibers.^[^ ^9^ ^]^ Piezoelectric tissues can convert mechanical loading into bioelectrical cues to regulate self‐healing,^[^ ^10^ ^]^ which inspires us to investigate the potential of piezoelectric heterojunctions as implants for tissue regeneration. In addition, the surfaces of the current implants are susceptible to bacterial adhesion, which can result in complications such as implant loosening and infection.^[^ ^11^ ^]^ This study utilized infected bone defects as a model and TiO_2_/Bi_2_WO_6_ as a heterojunction model to assess the potential of piezoelectric heterojunctions in regenerative medicine. The piezoelectric heterojunctions release electrical signals under mechanical stimulation from the cells and thus promote osteogenic differentiation (Scheme **1B‐b2**). Since ethylene glycol (EG) can reduce the oxygen ions in metallic oxide into oxygen and thus remove oxygen from the lattices to form oxygen vacancies,^[^ ^12^ ^]^ the heterojunctions can also produce EG‐induced oxygen vacancies, which introduces a new energy level in the bandgap and thus increase the capability for them to absorb near‐infrared (NIR) light and produce heat.

Consequently, their built‐in electric field (IEF) at the junction area also promotes the separation of NIR‐induced electron‐hole pairs and the transfer of the separated carriers from one semiconductor to another; such charge transfer allows the electrons and holes to react with oxygen and water, further facilitating the formation of reactive oxygen species (ROS). Both the heat and ROS collectively eliminate bacteria through photothermal and photodynamic therapy (Scheme 1B‐b1), respectively, allowing the piezoelectric heterojunction implants to kill implant‐associated bacteria while regenerating bone tissue to repair defects. The advantage of the photothermal and photodynamic therapy is the ease of triggering the therapy from outside the body by simply applying the tissue‐penetrating NIR light with a controlled power density.^[^ ^13^ ^]^ Although light‐based therapy is not specific to bacteria, we can shorten the irradiation time to the minimum duration (≈10 min) and direct light to only infected tissue, which can help reduce the impact on the normal cells of bone tissues within the irradiated area.

Bismuth tungstate (Bi_2_WO_6_), a narrow bandgap semiconductor material with piezoelectric properties,^[^ ^14^ ^]^ has good biocompatibility and has been applied to tissue repair.^[^ ^15^ ^]^ In this study, we utilized the piezoelectric properties of Bi_2_WO_6_ to construct a cellular electromechanical source, inducing in situ generation of electrical signals. Furthermore, we constructed tight, piezoelectric TiO_2_/Bi_2_WO_6_ heterojunction implants by growing Bi_2_WO_6_ nanocrystals on the TiO_2_ nanowires decorating the titanium implant surface through in situ hydrothermal growth (Scheme 1A). We found that the piezoelectric heterojunction implants could generate EG‐induced oxygen vacancies. These features contribute to the rapid generation of the aforementioned near‐infrared induced heat and ROS for efficient antimicrobial function through photothermal and photodynamic therapy, thus treating recurrent bacterial sensations in implants. When mouse bone marrow‐derived stem cells (mBMSCs) were cultured on the heterojunctions, their mechanical force stimulated the piezoelectric heterojunctions to generate an electrical signal to regulate bone regeneration. Thus, our piezoelectric heterojunctions can achieve efficient and dynamic antimicrobial function and promote osseointegration, demonstrating a clinically feasible idea for designing advanced biomedical implants. This strategy highlights the potential of piezoelectric heterojunctions in the field of regenerative medicine.

## 2 Results and Discussion

### 2.1 Cell‐Electromechanical Model Construction and Characterization

Bi_2_WO_6_ crystals, characterized by layered perovskite structures, exhibit piezoelectric properties stemming from the coherent deviation of W atoms from the center of the WO_6_ octahedron (Figure S1A, Supporting Information).^[^ ^14^, ^16^ ^]^ The nanostructured Bi_2_WO_6_, susceptible to deformation in the presence of mechanical force, holds significant piezoelectric potential for energy harvesting applications. We harnessed the piezoelectric potential difference generated by the interaction of piezoelectric heterojunctions with living cells to stimulate cell activity, constructing electromechanical models (Figure S1B,C, Supporting Information). To prove that a force applied to the heterojunctions by cells can stimulate the production of electrical signals, we fabricated an ideal model heterojunction by growing a layer of Bi_2_WO_6_ nanocrystal on the surface of the TiO_2_ substrate. Figures S2 and S3 (Supporting Information) depict the successful growth of Bi_2_WO_6_ nanocrystals on the surface of TiO_2_ substrate. Consequently, in situ hydrothermally synthesized TiO_2_/Bi_2_WO_6_ piezoelectric heterojunctions model on the implant surface were employed as cell culture substrates. Piezoelectric microscopy (PFM) revealed hysteresis phenomena in amplitude butterfly loops and phase hysteresis loops, confirming the piezoelectric properties of the TiO_2_/Bi_2_WO_6_ heterojunction model (Figure S4A, Supporting Information). The effective piezoelectric coefficient (d_33_) was determined as 8.122 pm V^−1^ through fitting the PFM amplitude loops (Figure S4B, Supporting Information), and the effective elastic constant was found to be 0.115 mN nm^−1^ for the TiO_2_/Bi_2_WO_6_ heterojunctions using the nanoindentation method (Figure S4C, Supporting Information). Considering the mechanical forces generated by cells within the nN range, including its gravity (≈0.03 nN) and traction (between 0.1 and 10 nN),^[^ ^17^ ^]^ notably, its gravity is insignificant compared to the traction generated by the cell and can be ignored in subsequent calculations. Ultimately, we applied Hooke's Law and the inverse piezoelectric effect equation to determine a piezoelectric potential ranging from 1–11 mV. To validate that the cellular force can induce the electric field generation due to the piezoelectric effect of the heterojunctions, COMSOL finite element simulation was employed. We found that different mechanical forces generated by cells could be converted into an electric potential ranging from 340 µV to 33 mV, consistent with experimental findings (Figure S4D,E, Supporting Information). The above results indicate that the intensity of the electrical signals generated by cellular mechanical force stimulation matches the range of physiological potentials required for bone regeneration.

To further experimentally verify the piezoelectric effect of the model heterojunctions on Ti implant surface, we employed a home‐made system (composed of Keithley‐7510 digital multimeter, a computerized data acquisition system, and a force transducer), we measured the voltage of the heterojunctions under a specific pressure (8 Pa) for multiple times (Figure S4F, Supporting Information). The results confirm the high force‐electric conversion performance of the piezoelectric heterojunctions on the implant surface, as the model stably produced an output voltage of ≈140 mV under constant pressure. The generated electrical signal is expected to promote new bone generation in the bone defect region.

To assess the feasibility of the TiO_2_/Bi_2_WO_6_ piezoelectric heterojunctions model as electrical stimulators of living cells, we evaluated biocompatibility through culturing mBMSCs on the heterojunctions. The piezoelectric heterojunctions model did not interfere with the cellular viability of mBMSCs (Figure S5, Supporting Information). Subsequently, we examined cell adhesion onto the piezoelectric heterojunctions. Confocal microscopy revealed spread and polygonal cellular focal adhesion on the heterojunction surface, indicating strong adhesion forces between the cells and the matrix (Figure S6, Supporting Information). The field emission scanning electron microscopy (FE‐SEM) further confirmed close contact between the plasma membrane of cells and the Bi_2_WO_6_ nanocrystal, with cellular protrusions firmly adhering to the heterojunctions (Figure S7, Supporting Information). The presence of electrical potential at the heterojunctions‐cell interface likely promoted the formation of pseudopods, enhancing cell attachment. These findings suggest that the piezoelectric potentials reaching mV levels can be generated using intrinsic cellular forces, offering in situ electrical stimulation of living cells to modulate their activity. This opens up the possibility of electrically induced self‐repair by the heterojunction implants.

### 2.2 Preparation and Characterization of Piezoelectric Heterojunctions

**Figure 1:**
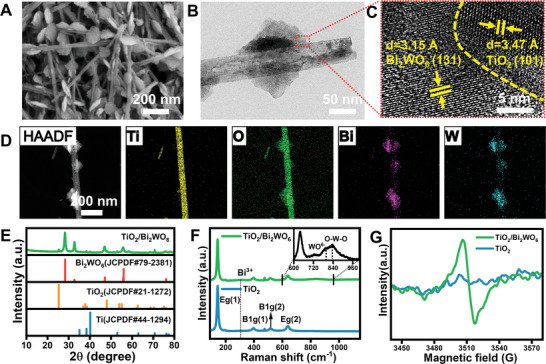
Preparation and characterization of piezoelectric heterojunctions (TiO_2_/Bi_2_WO_6_). A) FE‐SEM image, B) TEM image, and C) HRTEM images of red positions in Figure 2B of TiO_2_/Bi_2_WO_6_. D) HAADF‐STEM images of TiO_2_/Bi_2_WO_6_ and corresponding element mapping. E) XRD patterns of TiO_2_/Bi_2_WO_6._ F) Raman spectrum and G) EPR spectra of TiO_2_ and TiO_2_/Bi_2_WO_6_.

The piezoelectric heterojunctions (TiO_2_/Bi_2_WO_6_) were synthesized on the surface of titanium (Ti) implants using alkaline thermal treatment and in situ growth in EG. It should be noted that EG was used as a growth medium because EG could reduce the lattice oxygen ions into oxygen and thus facilitated the generation of oxygen vacancies to enhance photothermal therapy.^[^ ^18^ ^]^ As observed in Figure S8B (Supporting Information), FE‐SEM revealed a uniform distribution of TiO_2_ nanowires on the implant surface, each with a diameter of ≈30 nm, providing abundant nucleation sites for the growth of piezoelectric nanocrystals on the TiO_2_ nanowires. Furthermore, the structure effectively reduced the nucleation barriers and accelerated the adsorption and nucleation of Bi^3^^+^ and WO_4_^2−^ ions, resulting in the formation of tightly integrated heterojunction arrays (**Figure** **1A**). Transmission electron microscopy (TEM) further confirmed the in situ formation of piezoelectric heterojunctions along the nanowire (Figure 1B), illustrating that the size of the Bi_2_WO_6_ nanocrystals was ≈100 nm. High‐resolution TEM (HRTEM) images highlighted the distinguishable hetero‐interface between TiO_2_ and Bi_2_WO_6_, exhibiting lattice spacings consistent with the (101) plane of TiO_2_ and the (131) lattice plane of Bi_2_WO_6_ (Figure 1C). Additionally, the presence of four elements (Bi, W, O, and Ti) in TiO_2_/Bi_2_WO_6_ was confirmed by high‐angle annular dark‐field scanning transmission electron microscopy (HAADF‐STEM) and corresponding energy‐dispersive X‐ray spectroscopy (EDS) images (Figure 1D), with a number of distributed Bi and W elements on the smooth TiO_2_ nanowires surface. Monitoring the structural evolution of Bi_2_WO_6_ nanostructures on TiO_2_ nanowires over different reaction times revealed the increase in the size and elements Bi and W content of the piezoelectric heterojunctions with prolonged reaction time (Figure S8, Supporting Information), indicating successful construction of the TiO_2_/Bi_2_WO_6_. Therefore, in the subsequent study, we use the heterojunctions derived from the reaction time of 7 h.

X‐ray diffraction (XRD) patterns (Figure 1E) demonstrated that the diffraction peaks of TiO_2_/Bi_2_WO_6_ aligned well with anatase TiO_2_ (JCPDS No. 21–1272) and Bi_2_WO_6_ (JCPDS No. 79–2381). Raman spectra (Figure 1F) provided structural information related to the vibrational modes, with TiO_2_ exhibiting characteristic bands, and heterojunctions displaying characteristic Raman peaks of Bi_2_WO_6_ compounds. The presence of a non‐centrosymmetric distortion in the crystal structure,^[^ ^19^ ^]^ responsible for the piezoelectric property of Bi_2_WO_6_, was confirmed by the infrared‐active Raman peak at 825 cm^−1^. Furthermore, the surface chemical state of the TiO_2_/Bi_2_WO_6_ was investigated by X‐ray photoelectron spectroscopy (XPS) to explore the interaction between the two constituent semiconductors in the heterojunctions. The XPS survey spectra revealed the presence of Bi, W, Ti, and O elements in the TiO_2_/Bi_2_WO_6_ (Figure S9A, Supporting Information). The Ti 2p and W 4f peaks indicated Ti^4+^ and W species in the WO^6−^ octahedron, with a shift toward higher binding energy suggesting electron transfer from TiO_2_ to Bi_2_WO_6_ (Figure S9B,D, Supporting Information). High‐resolution XPS spectra of Bi elements on the TiO_2_/Bi_2_WO_6_ displayed doublet peaks corresponding to Bi^3+^ (Figure S9E, Supporting Information). Oxygen vacancies in the heterostructure were confirmed by the O 1s spectrum and EPR spectra (Figure 1G; Figure S9C, Supporting Information). The results collectively demonstrated intimate contacts and strong chemical interactions between TiO_2_ and Bi_2_WO_6_ in the piezoelectric heterostructure, leading to changes in the local environment and electron density of the elements, which facilitate the production of electron‐hole pairs. Thus, the successfully constructed arrays of piezoelectric heterojunctions sitting on the TiO_2_ nanowire offer the potential for light‐responsive electroactive multifunctional implants.

### 2.3 Optical and Photothermal Performance of Piezoelectric Heterojunctions

**Figure 2:**
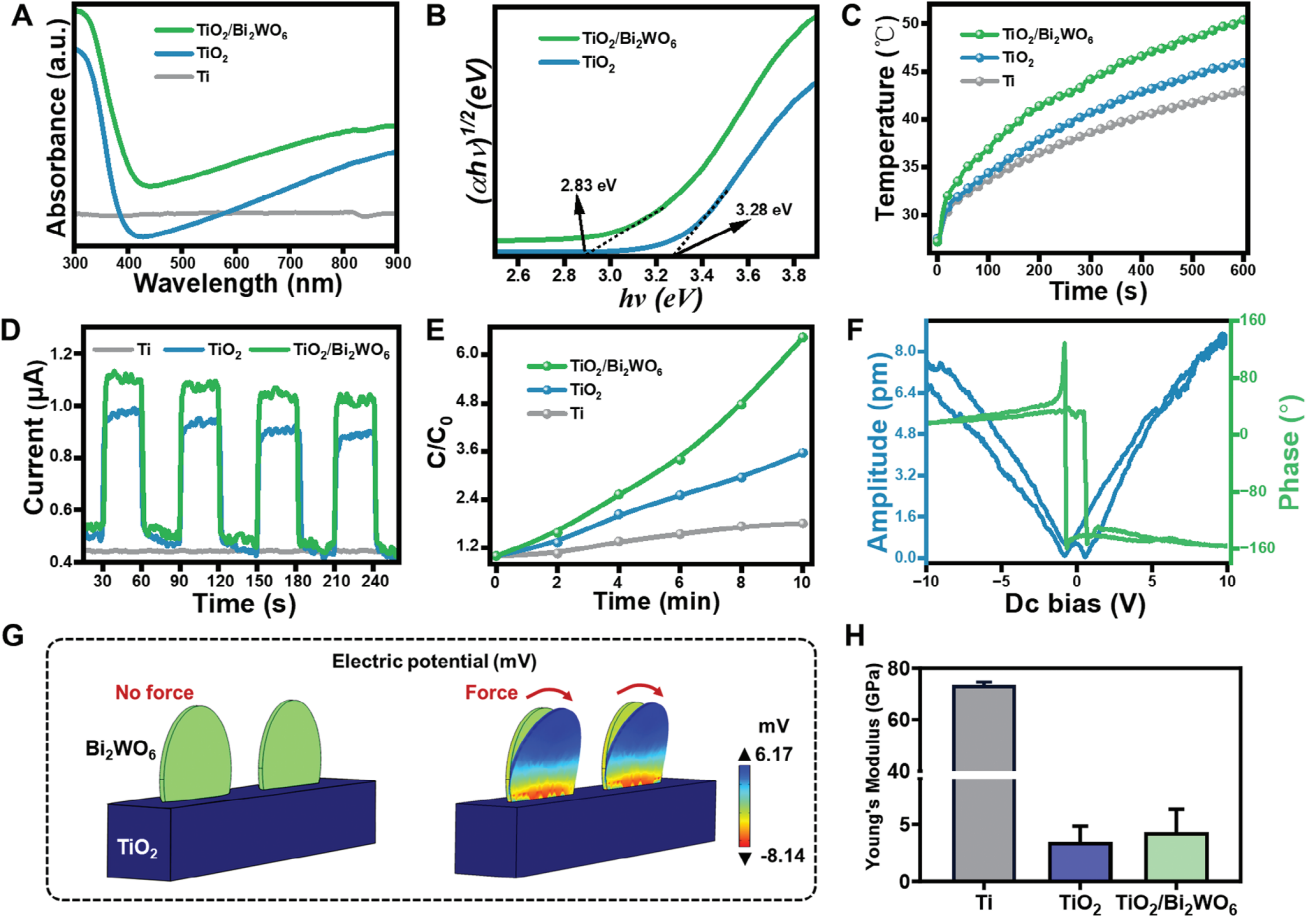
Optical, photothermal, and piezoelectric properties of TiO_2_/Bi_2_WO_6_ heterojunctions. A) The UV–vis DRS spectra and B) Plots of the (*αhv*)^1/2^ versus photon energy (*hv)*. C) Photothermal curves under NIR irradiation. D) Photocurrent under NIR irradiation (808 nm, 1 W cm^−2^, 10 min). E) ROS production under NIR irradiation as determined by DCFH‐DA. F) The local piezoelectric hysteresis loops of TiO_2_/Bi_2_WO_6_ heterojunctions. G) COMSOL simulation of electrical signals generated by TiO_2_/Bi_2_WO_6_ heterojunctions model when strained by cellular force. H) Young's modulus assay for titanium substrates (Ti), TiO_2_ nanowires on Ti substrates (TiO_2_), and TiO_2_/Bi_2_WO_6_ heterojunctions on Ti substrates.

**Figure** **2A** shows the UV–vis–NIR absorption spectra of all samples used to study the optical properties. The absorption edge of TiO_2_ and TiO_2_/Bi_2_WO_6_ is ≈390 and 470 nm, respectively, corresponding to the bandgap energy (*E_g_*) are 3.28 and 2.83 eV according to the Kubelka–Munk equation: *αhv = A(hv – E_g_)^n^* (Figure 2B).^[^ ^20^ ^]^ However, TiO_2_/Bi_2_WO_6_ showed stronger absorption activity in the visible‐NIR range, because of the heterojunctions and oxygen vacancies of the Bi_2_WO_6_ surface (Figure 1G). The photothermal properties of all samples were further evaluated (Figure 2C). The temperature of TiO_2_/Bi_2_WO_6_ increased and exceeded 50 °C compared to Ti and TiO_2_, indicating the promising capacity of TiO_2_/Bi_2_WO_6_ to efficiently convert NIR energy into thermal energy. At the same time, the evolution of the surface temperature at NIR irradiation times of 0, 2, 4, 6, 8, and 10 min was assessed using thermal images and photothermal stability (Figure S10, Supporting Information). Besides, to verify whether the photothermal effect of the heterojunction implants could be used for in situ hyperthermia under the tissue‐penetrating NIR light, a piece of the pork tissue with an overall thickness of 5 mm was chosen and placed on the implant (Figure S11A, Supporting Information). As shown in Figure S11B,C (Supporting Information), the temperature of all samples was reduced due to the light penetration of thick tissue, but the temperatures of the TiO_2_/Bi_2_WO_6_ could still be stably maintained at 45–50 °C. The above results proved that the NIR could successfully penetrate the thick layer of pork tissue and perform optical effects on implant surfaces.

### 2.4 Photoelectrochemical Performance of Piezoelectric Heterojunctions

Furthermore, photoelectrochemical experiments were conducted to investigate the separation of the electron‐hole pairs^[^ ^21^ ^]^ (and thus the generation of transient photocurrents) due to the IEF generated by the heterojunctions after NIR irradiation induced the formation of such pairs. The transient photocurrent spectra presented in Figure 2D reveal that the photocurrent generated by TiO_2_/Bi_2_WO_6_ is the highest among all specimens, indicating that the piezoelectric heterojunction promotes the rapid transfer of charge carriers, which is favorable for the utilization of NIR light. Electronic impedance spectroscopy (EIS) was employed to assess the charge separation and transfer ability of the TiO_2_/Bi_2_WO_6_ (Figure S12A, Supporting Information). As expected, the EIS curve of the TiO_2_/Bi_2_WO_6_ exhibits the smallest arc radius, indicating low charge transfer resistance and a fast interfacial charge transfer rate. Additionally, the photoluminescence (PL) spectra of TiO_2_/Bi_2_WO_6_ show the weaker fluorescent peak (Figure S12B, Supporting Information), suggesting that the heterojunctions can inhibit the recombination of the photogenerated electron and hole, consistent with the results of photoelectrochemical tests (Figure 2D). All these results demonstrate effective charge separation and transfer in the TiO_2_/Bi_2_WO_6_ implant, facilitating ROS production through the reaction of the separated electrons and holes with the oxygen and water, respectively.

Subsequently, 2′,7′‐dichlorofluorescein diacetate (DCFH‐DA) was employed to detect ROS production because DCFH‐DA can capture ROS rapidly to produce fluorescent 2′,7′‐dichlorofluorescein (DCF). As shown in Figure 2E, TiO_2_/Bi_2_WO_6_ exhibited the highest fluorescence intensity after NIR irradiation, indicating that the heterojunctions enhanced ROS production. Furthermore, electron spin resonance (ESR) was used to determine the types of ROS with the trapping agent being TEMP (2,2,6,6‐tetramethyl‐1‐piperidine, for singlet oxygen (^1^O_2_) in deionized water) and DMPO (5,5‐dimethyl‐1‐pyrroline N‐oxide, for superoxide radicals (•O_2_^−^) in methanol and hydroxyl radicals (•OH) in deionized water).^[^ ^22^ ^]^ These ROS were observed on the TiO_2_/Bi_2_WO_6_ under NIR irradiation (Figure S13, Supporting Information). These results indicate that the TiO_2_/Bi_2_WO_6_ heterojunctions promote ROS production, in addition to their strong photothermal conversion ability. This substantiates our belief that the TiO_2_/Bi_2_WO_6_ heterojunctions can inhibit bacterial infection and prevent recurrent implant infections through photothermal and photodynamic therapy in the NIR bio‐window.

### 2.5 Piezoelectric Properties of Piezoelectric Heterojunctions

To explore the electrical properties of the TiO_2_/Bi_2_WO_6_ heterojunctions, PFM was employed as reliable evidence for evaluating their piezoelectric characteristics. Figure 2F shows local piezoelectric hysteresis loops hysteresis and demonstrates that the phase angle produces a phase change in the range of 180° under ±10 V DC bias field. This evidence substantiates the occurrence of a local polarization transition process. Furthermore, the amplitude–voltage butterfly loops elucidate the local piezoelectric response of the piezoelectric heterojunction, consistent with reported literature results.^[^ ^23^ ^]^ In addition, the piezoelectric heterojunction significantly enhanced the piezoelectric properties compared to Bi_2_WO_6_ alone (Figure S14A,B, Supporting Information), which was attributed to the ability of the built‐in electric field of the piezoelectric heterojunctions to disrupt the central symmetry and induce polar symmetry,^[^ ^24^ ^]^ while the presence of the oxygen vacancy defects further disrupts the lattice symmetry of the material and promotes the formation of local polar regions.^[^ ^25^ ^]^ Furthermore, the impact of changes in cellular force on the surface potential of the TiO_2_/Bi_2_WO_6_ was confirmed through COMSOL finite element simulation. In Figure 2G, the mechanical force generated (10 nN) by the cell was demonstrated to be converted into a piezoelectric potential of 9.3 mV. A load force (0.1–10 nN) at the cell‐nanocrystal contact was enough to tilt them and drive in situ electrical signal generation (Figure S14C, Supporting Information).

Interestingly, the introduction of the piezoelectric heterojunctions also altered the surface mechanical properties of titanium implants. Nanoindentation tests revealed that the elastic modulus of TiO_2_/Bi_2_WO_6_ (≈5 GPa) was significantly lower than that of Ti implants (≈72 GPa) and closely aligned with that of natural bone tissue (≈3 to 20 GPa) (Figure 2H; Figure S15, Supporting Information). These results suggest that the constructed piezoelectric heterojunctions can self‐stimulate and regulate cellular activities, holding promise for inducing in situ electrical stimulation to promote bone regeneration.

### 2.6 Density Function Theory Calculations

**Figure 3:**
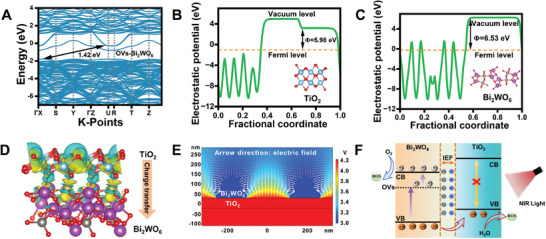
Density function theory calculations of TiO_2_ and Bi_2_WO_6_ were used to construct piezoelectric heterojunctions. A) The band structure of OVs‐Bi_2_WO_6_. The electrostatic potentials of B) TiO_2_ and C) Bi_2_WO_6_. D) Charge density distribution of TiO_2_/Bi_2_WO_6_ heterojunction (the yellow and blue color represents the electron accumulation and depletion region). E) Multiphysics field modeling to simulate the electric field of TiO_2_/Bi_2_WO_6_ heterojunction (arrows indicate electric field magnitude). F) Schematic illustration of ROS generation for TiO_2_/Bi_2_WO_6_ heterojunction under NIR irradiation.

To further elucidate the internal electronic structure and interactions between TiO_2_ and Bi_2_WO_6_, density function theory (DFT) was calculated to reveal the energy band and density of states (DOS) of TiO_2_ and Bi_2_WO_6_ (Figure S16, Supporting Information), revealing both as indirect bandgap semiconductors. This characteristic arises from the energy band structure, where the valence band maximum (VBM) and conduction band minimum (CBM) can be identified at specific points with high symmetry. The orbital component composition was analyzed using DOS, aligning with the literature results.^[^ ^26^ ^]^ Simultaneously, the energy band structure and DOS of the oxygen vacancy‐containing Bi_2_WO_6_ (OVs‐Bi_2_WO_6_) crystal structure were calculated to unveil the role of oxygen vacancies in the heterojunctions. As shown in **Figure** **3**A and Figure S17 (Supporting Information), the introduction of oxygen vacancies results in the appearance of an intermediate band in the middle of the Bi_2_WO_6_ bandgap.^[^ ^27^ ^]^ The DOS indicates that the intermediate band primarily originates from O 2p orbitals. The introduction of oxygen vacancies significantly reduces the photon energy required for electron leap in Bi_2_WO_6_, enhancing the efficiency of NIR light utilization.

Additionally, the work function (*Φ*), an essential parameter for interfacial charge transfer,^[^ ^28^ ^]^ was calculated for TiO_2_ and Bi_2_WO_6_, resulting in values of 5.98 and 6.53 eV, respectively (Figure 3B,C). Due to the smaller *Φ* of TiO_2_ compared to Bi_2_WO_6_, free electrons from TiO_2_ spontaneously flow to Bi_2_WO_6_ through the close contact of the heterojunction. This creates a positively charged electron depletion region on the TiO_2_ side and a negatively charged electron aggregation layer on the Bi_2_WO_6_ side, forming an IEF at the TiO_2_/Bi_2_WO_6_ heterojunction interface (Figure S18, Supporting Information). The charge density distribution of TiO_2_ and Bi_2_WO_6_ was simulated to more intuitively reflect the charge transfer process of the TiO_2_/Bi_2_WO_6_ heterojunction. As shown in Figure 3D, the Bi_2_WO_6_ side exhibits an electron aggregation region but experiences significant electron loss on the TiO_2_ side. This indicates the transfer of electrons from TiO_2_ to Bi_2_WO_6_ through the heterojunctions, consistent with the results of the work function analysis, theoretically confirming that the TiO_2_/Bi_2_WO_6_ heterojunction effectively promotes carrier transfer. Meanwhile, the spatial potential distribution and electric field direction of the piezoelectric heterojunctions were further simulated using COMSOL Multiphysics software (Figure 3E). The results indicate that the TiO_2_/Bi_2_WO_6_ interface generates an IEF with the direction pointing from TiO_2_ to Bi_2_WO_6_, which is favorable for the separation of photogenerated carriers. Based on the above discussion, a proposed mechanism is illustrated in Figure 3F. Under NIR irradiation, the TiO_2_/Bi_2_WO_6_ heterojunction facilitates photodynamic effects due to the interaction of energy bands and the functional interface, enhancing the production of ROS.

### 2.7 In Vitro Cell Cytocompatibility of Piezoelectric Heterojunctions

**Figure 4:**
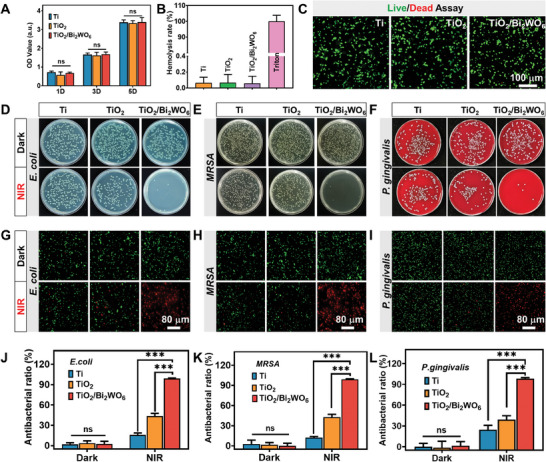
Biocompatibility and antibacterial assay of piezoelectric heterojunctions in vitro. A–C) Cell viability, hemolysis rate, and live/dead fluorescence staining of piezoelectric heterojunctions with mBMSCs in the dark. D–F) Spread plate, G–I) bacterial Live/dead fluorescence staining (red represent dead bacteria, green represent live bacteria), and J–L) antibacterial rate of *E. coli*, *MRSA*, and *P. gingivalis* with different samples under dark and NIR irradiation (808 nm, 1 W cm^−2^, 10 min). The data are represented in the format of means ± standard deviations (*n* = 3). ns represented *p* > 0.05, ^*^ *p* ≤ 0.05, ^**^ *p* < 0.01, and ^***^ *p* < 0.001.

The cytocompatibility of the TiO_2_/Bi_2_WO_6_ heterojunctions was investigated to validate their potential for biomedical applications. As depicted in **Figure** **4A**, there was no significant difference in the viability of mBMSCs cells among all implants, and those containing the lower concentrations of Bi and W elements also showed good bioactivity. Moreover, there was no significant phototoxicity observed in mBMSCs cells under NIR irradiation (Figure S19, Supporting Information), which was attributed to the fact that normal cells have a more effective protective mechanism against heat shock proteins.^[^ ^29^ ^]^ To further assess the biocompatibility of TiO_2_/Bi_2_WO_6_, we used erythrocytes from New Zealand rabbits to evaluate the hemolysis (Figure 4B). The samples demonstrated a hemolysis rate of less than 0.5%, different from the 100% hemolysis rate in the positive control group (Triton‐100X), indicating that the TiO_2_/Bi_2_WO_6_ heterojunctions exhibit no actual biotoxicity. Subsequently, we conducted live/dead fluorescence staining to visually study the cytotoxicity of different implants after 2 days of culture (Figure 4C). Compared with the other two samples, only a small amount of red staining was observed in the TiO_2_/Bi_2_WO_6_ group, consistent with the results of cell survival (Figure 4A). Therefore, it can be concluded that the TiO_2_/Bi_2_WO_6_ group exhibits good cytocompatibility and excellent photoresponsiveness, making it promising for applications in surgical implants.

### 2.8 In Vitro Antibacterial Activity of Piezoelectric Heterojunctions

Postoperative recurrent implant infections arise from re‐injury or infection of the tissues surrounding the implanted seed, with bacterial infections being a primary cause of recurrence. Hence, rapid eradication of bacterial infection is crucial, determining the subsequent healing ability of the bone. In this context, the antibacterial properties of the TiO_2_/Bi_2_WO_6_ heterojunctions under NIR irradiation were studied in vitro using three typical pathogenic bacteria (aerobes: *E. coli*, *MRSA*, and anaerobes: *P. gingivalis*). As depicted in Figure 4D–F, all implants exhibited nearly equal bacterial colonies after 10 min of incubation without NIR irradiation, indicating no significant antibacterial effect against *E. coli*, *MRSA*, and *P. gingivalis* under dark conditions. In contrast, under NIR irradiation, the TiO_2_/Bi_2_WO_6_ group showed almost no bacterial colonies compared to the Ti and TiO_2_ groups, demonstrating a favorable antibacterial ability toward *E. coli*, *MRSA*, and *P. gingivalis*. Furthermore, the TiO_2_/Bi_2_WO_6_ heterojunctions exhibited antibacterial rates of 99.03%, 99.11%, and 98.31% against *E. coli*, *MRSA*, and *P. gingivalis*, respectively (Figure 4J–L), highlighting the potential of the light‐responsive heterojunctions as a novel antibacterial material for implants.

Subsequent live/dead staining (Figure 4G–I) revealed predominantly red fluorescence in dead bacteria in the TiO_2_/Bi_2_WO_6_ heterojunction group under NIR irradiation. The other groups showed mainly live bacteria, similar to the antibacterial tests. Additionally, The surfaces of the *E. coli* and *MRSA* were smooth and regular in the control groups (dark‐ and light‐treated) (Figure S20, Supporting Information). The bacteria in the TiO_2_ group exhibited a slightly wrinkled surface, whereas those in the TiO_2_/Bi_2_WO_6_ group displayed wrinkled or ruptured cell membrane surfaces, indicating bacterial death (red arrows). Further validation of the damage caused by the TiO_2_/Bi_2_WO_6_ heterojunctions to *E. coli* and *MRSA* was confirmed through protein leakage experiments. No significant differences were observed in all samples under dark conditions, but after NIR irradiation, the TiO_2_/Bi_2_WO_6_ group exhibited more bacterial protein leakage than the other groups, suggesting the more dead bacteria (Figure S21, Supporting Information). In conclusion, the implanted heterojunction surface with dual photoresponsive synergy can effectively eliminate key bacteria associated with implant infections in a short period, presenting a potential solution for the treatment of recurrent implantation infections.

### 2.9 In Vitro Osteogenic Differentiation of Piezoelectric Heterojunctions

**Figure 5:**
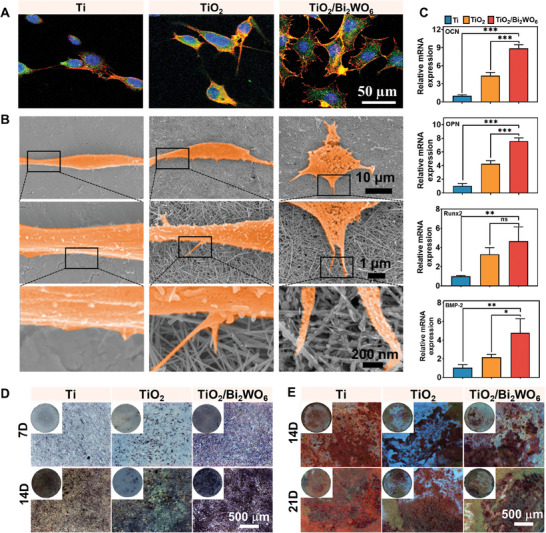
Osteogenic differentiation of piezoelectric heterojunctions in vitro. A) Immunofluorescence staining of vinculin (focal contacts, red) and F‐actin (stress fibres, green) was detected after 2 days of co‐culture with mBMSCs using CLSM. B) FE‐SEM morphology (different magnifications) of cells on different substrates including Ti substrates, TiO_2_ nanowires on Ti substrates, and TiO_2_/Bi_2_WO_6_ heterojunctions. C) Osteogenic‐related gene expression of OCN, OPN, Runx2, and BMP‐2 on the mBMSCs cultured on different sample surfaces after 7 days. D) ALP staining of mBMSC cultured on the different sample surfaces for 7 and 14 days (*n* = 3). E) ARS staining of mBMSC cultured on the different sample surfaces for 14 and 21 days (*n* = 3). The values indicated means ± standard deviations (*n* = 3): ns represented *p* > 0.05, ^*^ *p* ≤0.05, ^**^ *p* < 0.01, and ^***^ *p* < 0.001.

Previous studies have affirmed that cell adhesion is a crucial factor for biomaterials post‐implantation, playing a significant role in the generation of cell‐electromechanical induced in situ electrical signals, which directly influence the proliferation and differentiation behaviors of mBMSCs.^[^ ^17^, ^30^ ^]^ To investigate mBMSCs morphologies and adhesion behavior on various implant surfaces, FE‐SEM and confocal laser scanning microscopy (CLSM) observations were performed after co‐culturing for 2 days (**Figure** **5**A,B). The results demonstrated that mBMSCs seeded onto Ti substrates and the TiO_2_ group (TiO_2_ nanowires on Ti substrates) exhibited a spherical morphology without pseudopod extension. Surprisingly, TiO_2_/Bi_2_WO_6_ heterojunctions induced abundant F‐actin assembly and a more elongated spindle‐like morphology with a higher cell spreading area (Figure S22A, Supporting Information), aligning with the results of the cell‐electromechanical model above. Notably, the cellular pseudopods of mBMSCs on the TiO_2_/Bi_2_WO_6_ implant significantly increased, showcasing more spindle‐like and elongated morphologies, likely attributed to the micro‐nano structure and electrical properties of the piezoelectric heterojunction.

Furthermore, evaluating the osseointegration ability of electrical microenvironments on the TiO_2_/Bi_2_WO_6_ piezoelectric heterojunction surface can effectively reduce the risk of implant infection. The alkaline phosphatase (ALP) assay results in Figure 5D and Figure S22B (Supporting Information), on days 7 and 14 of co‐culture with mBMSCs on different implant surfaces, showed increased cellular ALP activity on the TiO_2_ surface compared to the Ti substrate, possibly due to the induction of osteogenic differentiation of mBMSCs cells by the bio‐nanowire structures. Importantly, the TiO_2_/Bi_2_WO_6_ group exhibited a higher ALP activity than the Ti and TiO_2_ groups, indicating that the coupling of in situ electrical signals stimulation and the bio‐micro‐ and nanostructure enhanced the early osteogenic differentiation. Moreover, we employed Alizarin‐red S (ARS) staining to evaluate calcium nodules, crucial biomarkers for evaluating late osteogenic differentiation on days 14 and 21. More calcium nodules were found on the TiO_2_/Bi_2_WO_6_ group than on the other two groups (Figure 5E), exhibiting the significantly increased mineralization of the extracellular matrix on the former. Additionally, the results of the quantitative analysis of ARS staining (Figure S22C, Supporting Information) further demonstrated that the functionalized interface of the TiO_2_/Bi_2_WO_6_ group significantly promotes osteogenic differentiation of mBMSCs. The TiO_2_/Bi_2_WO_6_ group presented significantly increased expression of osteogenic‐related genes (OCN, OPN, Runx2, and BMP‐2) compared to the Ti and TiO_2_ groups at 7 days (Figure 5C), indicating that TiO_2_/Bi_2_WO_6_ enhances the osteogenic differentiation of mBMSCs. Overall, the combination of the micro‐nano structure and in situ electrical stimulation of TiO_2_/Bi_2_WO_6_ heterojunction promotes the osseointegration capacity of mBMSCs in vitro.

### 2.10 Postoperative Staged Antibacterial and Osseointegration Evaluation in Vivo

**Figure 6:**
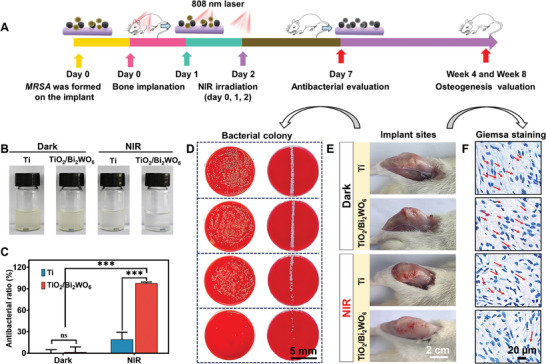
In vivo anti‐infection evaluation of the piezoelectric heterojunctions. A) Scheme of implantation surgery for assay in vivo. B) Photographs of media culture with the implants. C) In vivo antibacterial rate of piezoelectric heterojunctions in the presence or absence of NIR irradiation. D) Photographs of bacterial colony formation in contact with the implant. E) Photographs of the implant sites. F) Giemsa staining showing the spatial distribution of bacteria around the implants. The values indicated means ± standard deviations (*n* = 3): ^***^ *p* < 0.001.

Building upon the capability of light‐force‐electric coupling implants to combat pathogenic microorganisms and address poor osseointegration performance, a rat model was chosen to further evaluate the in vivo effects. We implanted the commercial biomedical Ti and TiO_2_/Bi_2_WO_6_ heterojunctions, both precontaminated with or without MRSA, into the bone defects. First, in the Ti +NIR and TiO_2_/Bi_2_WO_6_+NIR groups contaminated with MRSA, we used NIR light to irradiate the implanted sites on days 0, 1, and 2 after surgery (**Figure** **6** **A**). Additionally, NIR treatment was not applied to the implant group without infection to assess dynamic osseointegration performance (Figure S23, Supporting Information). After 7 days post‐implantation, we sacrificed the rats to determine the antibacterial properties of the implants and found that the Ti+NIR and TiO_2_/Bi_2_WO_6_+NIR groups reached an antibacterial rate of 19.18% and 97.81%, respectively (Figure 6C,D). We also found that the implant site of the Ti+NIR group presented inflammatory exudation and pus, which were reduced in the TiO_2_/Bi_2_WO_6_ group (Figure 6E). However, the TiO_2_/Bi_2_WO_6_+ NIR group did not have any inflammatory exudation and pus in the implantation sites due to the successful antibacterial therapy. Subsequently, we excised the heterojunction implants and rolled them on agar culture plates, followed by incubation in Luria−Bertani (LB) medium. We found the following order in terms of the colonies and LB turbidity: Ti+Dark > TiO_2_/Bi_2_WO_6_+ Dark > Ti+ NIR> TiO_2_/Bi_2_WO_6_+ NIR (Figure 6B,D). We also isolated implant infection tissues and assessed their infection status histologically. We discovered that the TiO_2_/Bi_2_WO_6_+ NIR group almost did not have any bacterial contamination compared to the other control groups (Figure 6F). Hence, the TiO_2_/Bi_2_WO_6_+ NIR group bears outstanding in vivo antibacterial capability, through a combination of PDT and PTT by the TiO_2_/Bi_2_WO_6_ heterojunctions.

**Figure 7:**
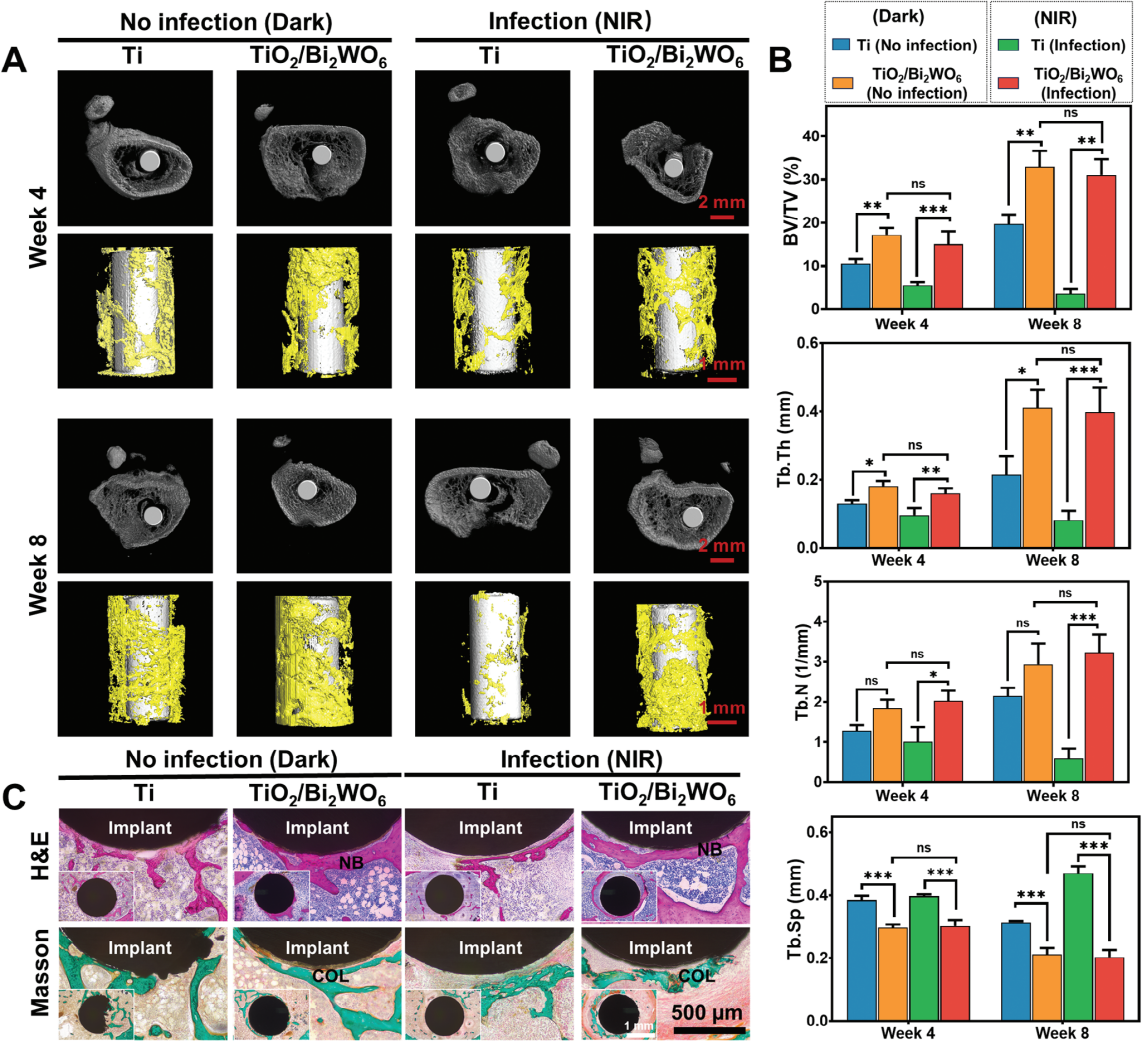
In vivo bone regeneration of piezoelectric heterojunction implants after 4 and 8 weeks. A) Micro‐CT images showing the bone repair around different implants. B) Quantitative micro‐CT data of BV/TV, Tb.Th, Tb.N, and Tb.Sp. C) H&E and Masson's Trichrome stating showing the new bone formation around the implant site at 8 weeks. One, two, and three asterisks represent a p value of less than 0.05, 0.01, and 0.001, respectively.

Continuing to explore cell‐electromechanical stimulation for osteogenic function after anti‐infection in vivo, we divided the rats into four groups: the Ti–No infection, the TiO_2_/Bi_2_WO_6_‐No infection, the Ti‐Infection + NIR, and the TiO_2_/Bi_2_WO_6_‐Infection + NIR (Figure S22, Supporting Information). After 4‐ and 8‐weeks post‐implantation, we sacrificed all of the animals to evaluate bone repair. As shown in **Figure** **7A**, the Ti–No infection group and Ti‐infection group observed limited newly formed bone (implant infection) due to severe bacterial infection or lack of bone formation capacity. In contrast, the TiO_2_/Bi_2_WO_6_‐infection group exhibited diminished bacterial infection in vivo following NIR irradiation. Meanwhile, the piezoelectric heterojunctions in the group of TiO_2_/Bi_2_WO_6_‐infection + NIR were conducive to the generation of new bone, with bone formation similar to that in the TiO_2_/Bi_2_WO_6_‐No infection group. By quantitatively analyzing the micro‐computed tomography (micro‐CT) data, we found that the groups of TiO_2_/Bi_2_WO_6_‐No infection and TiO_2_/Bi_2_WO_6_‐Infection+ NIR almost presented the highest values of the following parameters, including trabecular thickness (Tb. Th), trabecular number (Tb. N), bone volume/total volume (BV/TV), and trabecular separation (Tb. Sp) (Figure 7B). This result implies that the photoresponsive piezoelectric heterojunction can temporally regulate antimicrobial and osteogenic behaviors in vivo. Moreover, the implant‐containing femur was examined for new bone tissue formation and implant contact with bone using H&E and Masson's trichrome staining. As shown in Figure 7C, for the Ti–No infection group and the Ti‐Infection + NIR group, there was almost no new bone between the implant and native bone due to severe bacterial infection or the lack of an electroactive microenvironment after 8 weeks, resulting in deterioration of osteogenesis. However, the TiO_2_/Bi_2_WO_6_‐No infection group and the TiO_2_/Bi_2_WO_6_‐Infection + NIR group showed a large amount of new bone tissue formation, and the edges of the bone defects on the implant were connected to connective fiber tissue, suggesting that the bone tissue was integrated with the implant and continued to grow. Additionally, Masson staining images showed a large amount of collagen formation around the implants in the TiO_2_/Bi_2_WO_6_ group. At the same time, the rats in all experimental groups were sacrificed, and their major organs (heart, liver, spleen, lungs, and kidneys) were taken for histological analysis (Figure S24, Supporting Information). The results did not reveal the obvious difference between the TiO_2_/Bi_2_WO_6_‐Infection + NIR group and the uninfected group without treatment, indicating satisfactory biosafety of the heterojunctions.

### 2.11 The Molecular Mechanism of Osteogenesis Enhanced by Piezoelectric Heterojunctions

**Figure 8:**
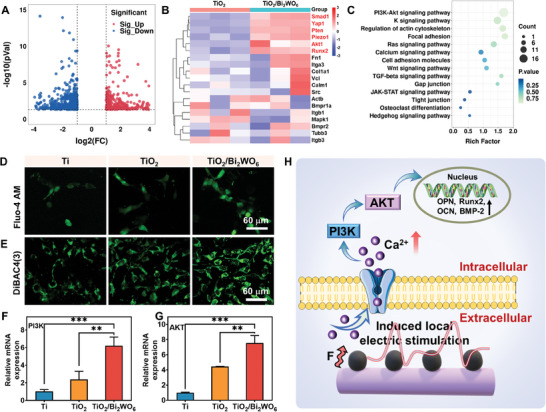
The molecular mechanisms for the piezoelectric heterojunctions to enhance osteogenesis. A) Volcano plots of differentially expressed mRNAs selected from RNA sequencing data set of TiO_2_ versus TiO_2_/Bi_2_WO_6_ group. B) Heatmap of differentially upregulated cytokines associated with osteogenesis activity of TiO_2_ versus TiO_2_/Bi_2_WO_6_ group. C) The KEGG functional enrichment analysis of differentially expressed genes between TiO_2_ versus TiO_2_/Bi_2_WO_6_ group. D) Intracellular Ca^2+^ staining and E) cell membrane potential imagene expression in mBMSCs on day 7. H) Scheme of osteogenic differentiation mechanism of mBMSCs co‐cultured with piezoelectric heterojunctions. The data represented means ± standard deviations (*n* = 3) with ns indicating a *p* value of higher than 0.05. *p ≤ 0.05, **p < 0.01, and ***p < 0.001.

Electromechanical signals generated based on piezoelectric heterojunctions can directly influence cell adhesion and spreading behavior. We further used RNA‐sequencing (RNA‐seq) to evaluate the piezoelectric heterojunction‐enhanced gene expression in order to gain a better understanding of the molecular mechanism. The principal component analysis (PCA) (Figure S25A, Supporting Information) clearly indicated that the TiO_2_/Bi_2_WO_6_ heterojunctions caused the differences in gene expression compared to other groups, further confirming the role of the cell‐force‐induced electric signals in osteogenesis. We found that 841 genes (340 up‐regulated and 501 down‐regulated) in the TiO_2_/Bi_2_WO_6_ heterojunction group were very different from those in the TiO_2_ group by analyzing the volcano plot (**Figure** **8**A). From the Venn diagram, we determined that 170 differentially expressed genes (DEGs) are shared between the TiO_2_‐Ti and TiO_2_/Bi_2_WO_6_‐TiO_2_ groups (Figure S25B, Supporting Information). The results suggest that the application of heterojunction‐produced electrical signals (generated by cell mechanical forces) to the cells causes the gene expression to be significantly different between different groups, indicating the different molecular mechanisms in enhancing osteogenesis under the guidance of the piezoelectric heterojunctions.

Judged from the heatmap, we determined that the piezoelectric heterojunctions widen the difference in gene expression (Figure 8B; S25C, Supporting Information). Among these genes, the calmodulin (Calm), an important transporter in Ca^2+^ transport was noticed. Being positioned in the downstream of the calcium‐signaling pathway, it can bind Ca^2+^ in the cells.^[^ ^31^ ^]^ More importantly, the TiO_2_/Bi_2_WO_6_ heterojunctions exhibit significantly upregulated expression of Itga3, piezo1, and Yap1, which are involved in cell–material interactions and downstream cell fate commitment. We believe that the TiO_2_/Bi_2_WO_6_ heterojunctions can promote the adhesion and spreading of mBMSCs with the genes upregulated to enhance PI3K‐AKT signaling (Akt1 and Src), in agreement with the reported electrical activation of Src to mediate wound healing by means of PI(3)Kγ.^[^ ^32^ ^]^ It is now known that the PI3K‐AKT signaling pathway regulates the process of bone formation and remodeling.^[^ ^33^ ^]^ These signaling pathways were also significantly enriched for differentially expressed genes in KEEG analysis, and the differences were even more pronounced (Figure 8C; Figure S25D, Supporting Information).

Having established that the electric microenvironment plays a pivotal role in mBMSCs toward osteogenesis, we next investigated the changes in Ca^2+^ levels in mBMSCs using the fluorescent probe Fluo‐4 AM to verify that electrical signals induced the calcium enrichment. As shown in Figure 8D and Figure S26A (Supporting Information), the TiO_2_/Bi_2_WO_6_ piezoelectric heterojunctions could induce calcium influx, increasing the intracellular Ca^2+^ concentrations (correlated with the green fluorescent signal). This result suggests that the electrical signals, produced due to the cell mechanical force acting on the heterojunctions, elevated the calcium influx. It is well‐known that as a secondary messenger Ca^2+^ participates in several signaling pathways,^[^ ^34^ ^]^ and electrical signal stimulation can induce an increase in intracellular Ca^2+^ concentration in osteoblasts.^[^ ^35^ ^]^ Likewise, the cell membrane potential observed followed a similar trend of changes due to the stimulation of electrical signals on the surface of heterojunctions (Figure 8E; Figure S26B, Supporting Information). The above research indicates that electrical signal activation of the phosphatidylinositol 3‐kinase (PI3K) and protein kinase B (AKT) pathway is related to osteogenic differentiation. As shown in Figures 8F,G, the PI3K/AKT gene expression in cells co‐cultured on the TiO_2_/Bi_2_WO_6_ group had significantly enhanced expression compared to the TiO_2_ and Ti groups using qPCR analysis, confirming that electrical signals from heterojunctions can be transduced into cells via the PI3K‐AKT pathway. Besides, YAP is a key mechanotransduction regulator of cells in response to mechanical and topographical stimulation.^[^ ^36^ ^]^ We assessed YAP gene expression using qPCR and found that the gene expression in the TiO_2_/Bi_2_WO_6_ group increased 1.88‐fold versus 2.42‐fold compared to that in the Ti and TiO_2_ groups (Figure S26C, Supporting Information). We therefore proposed a mechanism shown in Figure 8H. This suggests that the TiO_2_/Bi_2_WO_6_ piezoelectric heterojunctions can induce biomechanical signal transduction of mBMSCs and further affect cellular force‐electric conversion capacity. These results suggest that piezoelectric heterojunctions can promote osteogenic differentiation and osseointegration of mBMSCs through nanostructure and localized electrical microenvironments, elucidating a potential mechanism of cell‐electromechanical signals‐mediated osteogenesis.

The above results indicate that our piezoelectric heterojunctions can exhibit excellent optical‐force‐electrical coupling functional properties to promote the repair of infected bone defects by utilizing engineered oxygen vacancies and built‐in electric fields, compared with reported non‐heterojunction piezoelectric materials such as BaTiO_3_, CoFe_2_O_4_@BaTiO_3_/P (VDF‐TrFE) composite membranes, and poly(l‐lactic acid).^[^ ^37^ ^]^ On the one hand, the mechanical force exerted in situ by surface‐attached BMSCs was utilized to stimulate the generation of electrical signals, avoiding tissue damage from exogenous stimulation. On the other hand, under the excitation of near‐infrared light (NIR), the piezoelectric heterojunction generates ROS and heat within a short period of time (≈10 min), which synergizes the photodynamic and photothermal properties to destroy bacteria, creating a sterile microenvironment for bone tissue regeneration.

## 3 Conclusion

In conclusion, we have developed a cell‐electromechanical model and designed piezoelectric heterojunction implants for antimicrobial therapy and osteogenesis. Upon NIR light stimulation, the heterojunctions produce heat by increasing the absorption at the NIR window (through generating oxygen vacancies) and generate ROS through charge transfer enabled by IEF. Hence, the heterojunctions can eradicate bacteria through photothermal and photodynamic therapy, demonstrating significant potential for treating bacterial infections in recurrent implant infections. Importantly, the cells growing on the heterojunctions can apply mechanical forces onto them, generating electric signals to mediate cell fates and thus promoting bone regeneration and osseointegration. Moreover, the piezoelectric heterojunction implant, with biomechanical electrical stimulation, activates the calcium enrichment pathway and the PI3K‐AKT signaling pathway. In vivo, experiments in a model of infected bone defects validate the ability of light‐responsive electroactive implants to temporally prevent bacterial infection and promote bone tissue regeneration. The developed piezoelectric heterojunctions, responsive to light and cellular force electrical stimulation, offer a novel approach to repairing recurrent infected bone defects and present innovative strategies for treating damaged infected tissues by re‐establishing the electrical microenvironment and eradicating bacterial infection.

## 4 Experimental Section

### Materials

Titanium was purchased from Qichen New Materia Technology Co., Ltd (Baoji, China). HNO_3_ (AR, 65.0–68.0%) and HCl (AR, 36.0–38.0%) were purchased from Guangzhou Reagent Factory. HF (AR, ≥40.0%), NaOH (ACS, >97%), Bi(NO_3_)_3_·5H_2_O (AR, 99.0%), Na_2_WO_4_·2H_2_O (AR, 99%) and Ethylene glycol (AR, 98.0%) were obtained from Aladdin Co., Ltd. (Shanghai, China). The water used in all experiments was de‐ionized (DI).

### Preparation of TiO_2_/Bi_2_WO_6_ Piezoelectric Heterogeneous on Titanium Implant

TiO_2_ nanowires were successfully constructed using an alkaline activation method on the titanium implant surface. Before alkaline activation, the titanium is cleaned with acetone, ethanol, and DI under ultrasonic action for 15–20 min, and then acid‐etched with a certain proportion of HNO_3_ and HF solution to remove the oxidized layer on the surface of the implant. Next, the titanium substrates were alkali thermally treated with 2 m NaOH at 200 °C oven for 18 h. After hydrothermal treatment, the implants were treated in 0.6 m HCl solution for 1 h and subsequently heat‐treated in air at 550 °C.

Piezoelectric heterojunctions were prepared through a solvothermal method. First, Bi(NO_3_)_3_·5H_2_O and Na_2_WO_4_·2H_2_O with a molar ratio (Bi/W) of 2: 1 were mixed in 60 mL of EG so as to form the solution. Then, the alkali heat‐treated titanium implant was immersed in Bi_2_WO_6_ precursor solution, and hydrothermal heated to 160 °C for 3, 7, and 15 h. After the reaction was finished and cooled naturally.

### Preparation of TiO_2_/Bi_2_WO_6_ Heterogeneous Model on Titanium Implant

Titanium plates were ultrasonically cleaned with acetone, ethanol, and deionized water for 15–20 min, followed by air‐heat treatment at 550 °C. Finally, the surface of the titanium implant containing titanium dioxide was added to the mixed solution with 1 mm Bi(NO_3_)_3_·5H_2_O and 0.5 mm Na_2_WO_4_·2H_2_O. The resultant mixture was then added to a 100 mL Teflon‐lined autoclave and heated to 160 °C for 20 h. After reactions, the collected Ti plates were dried at 60 °C for 12 h.

### Characterization of the Piezoelectric Heterogeneous on Titanium Implant

XRD patterns were implemented on an Empyrean instrument operating with Cu‐Ka source (PANalytical, the Netherlands). Morphological characterization of the piezoelectric heterostructures was performed by field emission scanning electron microscopy (FE‐SEM) using a Merlin instrument from Carl Zeiss, Germany, and transmission electron microscopy (TEM) utilizing a Talos F200X microscope from Thermo Fisher operated. High‐resolution TEM (HRTEM) images were collected using a JEOL JEM‐ARF200F microscope with spherical aberration correction. Raman spectroscopy was executed on a Thermo DXR2xi Raman spectrometer. The element composition of the piezoelectric heterogeneous was characterized by XPS (ESCALAB XI+, Thermo Scientific), with binding energies calibrated against impurity carbon (C 1s = 284.8 eV). UV–vis–NIR) absorption spectra were measured by a UV‐3600 Plus spectrophotometer from Shimadzu, Japan. The force‐electric properties of piezoelectric heterojunctions were evaluated using a Keithley General Purpose Digital Meter (DMM7510) with applied constant weights. Electron spin resonance (ESR) spectra were captured at room temperature with a Bruker ELEXSYS‐II spectrometer. An Edinburgh Instruments FLS980 spectrometer was used to obtain the photoluminescence (PL) spectra under 233 nm excitation. The mechanical stability of the implant surface was evaluated by nanoindentation testing (Anton Paar, TTX‐NHT3, Switzerland). Piezoelectric properties were investigated via atomic force microscopy (AFM), employing the PFM mode on a Bruker Multimode 8 system from Germany. COMSOL was performed to simulate the finite element of the piezoelectric potential generated by the force exerted by the cells on the piezoelectric material.

### Photoelectrochemical Measurements

Electrochemical characterization of the samples was conducted using an electrochemical workstation (ZAHNER, Germany) equipped with a conventional three‐electrode system.^[^ ^38^ ^]^ All measurements were performed in Na_2_SO_4_ electrolyte solution at ambient temperature. Photocurrent measurements were executed under irradiation at a wavelength of 808 nm. Transient photocurrent responses were recorded both in the presence and absence of NIR irradiation. Electrochemical impedance spectroscopy (EIS) was done over a frequency range from 0.01 Hz to 10 kHz, with an applied amplitude of 5 mV.

### Detection of Reactive Oxygen Species (ROS) generation

An oxidation‐sensitive fluorescent dye, Dichlorodihydrofluorescein diacetate (DCFH‐DA, KeyGEM, China), was adopted to confirm the ROS production. Samples were immersed in a 48‐well plate containing DCFH‐DA solution and then irradiated with NIR light (808 nm, 10 min, 1.0 W cm^−2^). The dye was consumed every 2 min to determine the ROS content. All measurements were carried out in a dark environment. The different types of active oxygen produced by TiO_2_/Bi_2_WO_6_ heterojunction on titanium implants were tested using an electron paramagnetic resonance (EPR, EMXPlus 10/12, Bruker) spectrometer. TEMP was used as a singlet oxygen (^1^O_2_) trapping agent. The generation of ·O_2_^−^ and ·OH under NIR irradiation was tested by their characteristic reaction with DMPO.

### Photothermal Effects

The photothermal conversion was assessed using a thermal imaging camera (Fotric‐220) at NIR irradiation. The heating–cooling cycle curve of TiO_2_/Bi_2_WO_6_ was obtained under 808 nm irradiation. The photothermal conversion ability was further investigated by allowing the NIR light to pass through porcine skin with a thickness of ≈5 mm.

### DFT Calculation

To elucidate the heterojunction interface properties of TiO_2_ and Bi_2_WO_6_, DFT was simulated using the Vienna ab initio simulation package (VASP).^[^ ^39^ ^]^ A generalized gradient correlation function, generalized gradient approximation (GGA) exchange, and correlation functions in the Perdew–Burke–Ernzerh of (PBE) scheme were utilized. The electronic structure of TiO_2_ and Bi_2_WO_6_ semiconductors was characterized through the band structure and density of states (DOS) plots via first‐principles DFT calculations. 520 eV was set as a plane wave cutoff energy, and the Brillouin zone was sampled with a 2 × 4 × 2, 5 × 5 × 3, and 2 × 2 × 1 Monkhorst Pack grid for TiO_2_ and Bi_2_WO_6_ respectively. All atoms of TiO_2_ and Bi_2_WO_6_ were fully relaxed to their equilibrium positions, with an energy convergence of 1 × 10^−5^ eV and a force criterion of less than 0.01 eV/Å.

### Antibacterial Assessment in Vitro

*Staphylococcus aureus* (*MRSA*, ATCC25923), *Escherichia coli* (*E. coli*, ATCC25922), and *Porphyromonas gingivalis* (*P. gingivalis*, ATCC 33 277) were provided by ATCC and were used in light‐responsive antibacterial experiments in vitro. The spread plate method was used to determine the antibacterial rate. Initially, 400 µL of bacterial suspension (1 × 10^6^ CFU mL^−1^) was incubated with different samples in 48‐well plates and then exposed to NIR light (10 min). The obtained bacteria suspension was diluted 400 times, spread onto agar plates, and then incubated at 37 °C. Moreover, the group without light irradiation was used as the control. Subsequently, the number of viable colony‐forming units (CFUs) was calculated in the different groups. The corresponding antimicrobial rate was calculated as follows:

(1) $$\mathrm{Antibacterial}\;\mathrm{ratio}\;(\%)=\frac{CFU_{\left(Blank\right)}-CFU_{\left(sample\right)}}{CFU_{\left(Blank\right)}}\times 100\%$$

### Bacterial Morphology

SEM was utilized to characterize bacteria on the various titanium surfaces. Initially, the bacteria were fixed with 2.5% paraformaldehyde and subsequently dehydrated using a conventional ethanol gradient (30%, 50%, 70%, 80%, 90%, and 100%) before being dried. The prepared bacterial samples were then inspected using SEM for morphology analysis.

### Bacterial Live/Dead Staining

The bacteria after treatments were stained using a mixture of SYTO9 and PI from the LIVE/DEAD BacLight Bacterial Viability Kit (ThermoFisher, USA). After a 15‐min incubation period, phosphate‐buffered saline (PBS) was used to rinse the samples three times and imaged them under an inverted fluorescence microscope (IFM, Axio Observer.7).

### Bacterial Protein Leakage Quantification

The protein leakage was quantified from bacteria using a bicinchoninic acid (BCA, Beyotime, China) assay. After the sterilization experiment, the bacterial solution was diluted and mixed with sterile PBS at a 1: 1 volume ratio. The mixture was then centrifuged (10 000 rpm) and collected the supernatant, to which the BCA reagent was added. The mixture was then incubated at 37 °C for 30 min. Relative protein leakage was calculated by spectrophotometrically measuring OD_562_ _nm_ using a microplate reader (Cytation 5, BioTek, USA).

### Cell Culture

mBMSCs (ZQ0465; Shanghai) were used to assess cytocompatibility and osteoblastic differentiation. The mBMSCs were maintained in culture flasks using Dulbecco's Modified Eagle Medium (DMEM, Gibco, USA). The medium was supplemented with 10% fetal bovine serum (Sciencell, USA) and 1% penicillin–streptomycin solution (Sciencell, USA). Cells were incubated in an incubator (5% CO_2_, 37 °C) with the medium changed every 2 h to maintain optimal cell growth conditions.

### Cytotoxicity Evaluation

The cytotoxicity of the samples was evaluated using the CCK‐8 assay (Dojindo, Japan). A suspension of mBMSCs was seeded onto sterilized samples at 5000 cells/well. After different incubation periods (1, 3, and 5 days), DMEM was removed, 150 µL of a diluted CCK‐8 solution (CCK‐8 to DMEM ratio of 1: 10) was added to each well, and the plates were incubated at 37 °C for 2 h. Subsequently, CCK‐8 assay was used to determine the cell viability after exposure to NIR light for 10 min.

### Cell Live/Dead Staining

Live/dead staining was conducted using the fluorescent dyes calcein‐AM (Invitrogen, Japan) and propidium iodide (PI, Invitrogen, Japan). Following a 2 days co‐culture with the samples, cells were incubated with both PI and calcein‐AM for 30 minutes at 37 °C. Subsequently, a fluorescence microscope (Olympus IX 71, Olympus, Japan) was used to image the cells.

### Cell Fluorescence and Adhesion Analysis

The co‐culturing of mBMSCs and samples was conducted for 2 days. Subsequently, the samples containing mBMSCs were rinsed and fixed the cells with 4% formaldehyde (AR, Tianjin) for 30 min. The cells were then incubated with Vinculin Rabbit Monoclonal Antibody (Beyotime, China) primary antibody at 4 °C for 12 h and treated them with Alexa Fluor 488 goat anti‐mouse IgG1 (Beyotime, China) for 30 min in the dark. Actin‐Tracker Green (YiSen, Shanghai) was used to stain the cells for 1.5 h in the dark and DAPI (Beyotime, China) to stain cell nuclei for 5 min. The cellular morphology and distribution were visualized using a confocal laser scanning microscope (CLSM, Leica, Germany).

### Cell Observation by the FE‐SEM

Piezoelectric heterojunction implants were placed into 48‐well plates and co‐cultured with mBMSCs (5000 cells/well) for 2 days. After washing with PBS, the cells were fixed with glutaraldehyde. After dehydration in ethanol, the cells were observed under FE‐SEM.

### Intracellular Ca^2+^ Concentration and Membrane Potential Detection

After two days of co‐culturing the cells with the samples, Fluo‐4 AM solution and DiSBAC2(3) solution (Beyotime, China) were added to the samples to assess the intracellular Ca^2+^ concentration and cell membrane potential. After incubation at 37 °C for 30 min, the cells were imaged using CLSM.

### Osteogenic Differentiation

For the quantitative ALP activity assay, mBMSCs cultured on various titanium substrates for 7 and 14 days were rinsed with PBS and then incubated with cell lysis buffer (without inhibitors) designed for Western blotting and immunoprecipitation (Beyotime, China). The supernatant was collected, and 5 µL of phenolic standard solution was added to a 96‐well plate. A p‐nitrophenyl phosphate assay kit was used to quantify the ALP activity and employed a BCA assay (Beyotime, China) to determine total protein content. The optical densities (OD) were measured at 490 and 570 nm. Concurrently, staining reactions were performed using an alkaline phosphatase chromogenic kit. To assess mineralization, calcium nodules produced by the cells were stained with ARS (KGA363, KeyGEN BioTECH).

### RNA Sequencing and Mechanism Validation

Total RNA was obtained from mBMSCs using TRIzol reagent (ThermoFisher, USA) after 1 day of co‐cultivation with Ti, TiO_2,_ and TiO_2_/Bi_2_WO_6_ piezoelectric heterojunctions. Differential expression analysis was performed using the Must‐Seq platform.

### Quantitative Real‐Time Polymerase Chain Reaction

The expression of genes associated with osteogenesis was analyzed by quantitative real‐time PCR (qRT‐PCR), using the primers for OCN, OPN, Runx2, BMP‐2, PI3K, AKT, and YAP (**Table** **1**) and GAPDH as a housekeeping gene (Magen, Guangzhou).

**Table 1:** Primer sequences of genes related with osteogenic.

Primer	Forward (5′‐3′)	Reverse (5′‐3′)
*OPN*	TGGCTGAATTCTGAGGGACTAAC	TTCTGAGATGGGTCAGGCAC
*Runx2*	GGGAACCAAGAAGGCACAGA	ACTTGGTGCAGAGTTCAGGG
*OCN*	CCCTGAGTCTGACAAAGCCTTCA	AGATGCGTTTGTAGGCGGTC
*BMP‐2*	CCAGACGATCATGCAGCTAC	TCAACTCAAATTCGCTGAGGAC
*PI3K*	GCAACTCCTGGACTGCAACT	CAGCGCACTGTCATGGTATG
*Akt1*	ATGAACGACGTAGCCATTGTG	TTGTAGCCAATAAAGGTGCCAT
*YAP*	GACGCTGATGAACTCTGC	GATGTGGTCTTGTTCTTATGGT
*GAPDH*	GGCAAGTTCAACGGCACAGT	GCCAGTAGACTCCACGACAT

### Animal Implantation Surgery

A rat femoral prosthesis implant infection model was established. All animal surgeries were approved by the Experimental Animal Welfare and Ethics Committee of Zhuhai BesTest Bio‐Tech Co, Ltd (IAC202212014(B)). 30 SPF‐grade adult male SD rats were randomly assigned to six groups: Ti‐No infection, Ti‐Infection, Ti‐Infection + NIR, TiO_2_/Bi_2_WO_6_‐No infection, TiO_2_/Bi_2_WO_6_‐Infection, and TiO_2_/Bi_2_WO_6_‐Infection + NIR. Rats were anesthetized by injecting a combination of Sutent and Somnolensin. An incision was made at the distal end of the femur using a scalpel, followed by drilling a cylindrical wound with a diameter of ≈1.5 mm at the incision using a surgical drill. A sample soaked in a 10^7^ CFU mL^−1^ MRSA bacterial suspension for 1 h was implanted into the defect, which was then closed with bone wax and sutured layer by layer. After suturing, the surgical site of infected animals was irradiated with NIR light (10 min) for three days. All animals were fed with a standard diet, and the bone tissues were collected at the time points of weeks 1, 4, and 8 for further exploration.

### Antibacterial Analysis in Vivo

Several SD rats were randomly selected from each group to evaluate the femur, implant, and surrounding tissues for infection one week after surgery.

### Micro‐CT Analysis

Undecalcified specimens from each group were fixed using 4% paraformaldehyde and subsequently scanned using micro‐CT (XTV160H, UK). A region within 480 µm from the implant surface was designated as the volume of interest (VOI) for quantitative analysis.

### Histological Analysis

Femurs containing implants from different experimental groups were subjected to decalcification. Histological evaluation of new bone tissue formation was performed using Masson's trichrome and H&E staining. Initially, the femurs were fixed in 4% formaldehyde for a period of 3 days, followed by decalcification in an ethylenediaminetetraacetic acid (EDTA) solution for 28 days. The decalcified implants were then dehydrated, paraffin‐embedded, sectioned, stained (H&E and Masson, Biosharp, Beijing), and imaged using the 3DHISTECH Case Center (P250 FLASH).

### Biocompatibility in vivo

To further assess in vivo biocompatibility, major organs (heart, liver, spleen, lung, and kidney) in the Ti and TiO_2_/Bi_2_WO_6_ groups were isolated, H&E stained, and examined under the 3DHISTECH Case Center (P250 FLASH) microscope.

### Statistical Analysis

The statistical analysis was performed using Prism 10. All results were presented as means ± standard deviation (SD) from at least triplicate tests (*n* ≥ 3). Statistical differences between multiple groups were determined using one‐way analysis of variance (ANOVA). *p* < 0.05 was considered statistically significant (^*^ *p* < 0.05, ^**^ *p* < 0.01, and ^***^ *p* < 0.001).

## Conflict of Interest

The authors declare no conflict of interest.

## Supporting information



Supporting Information

## Data Availability

The data that support the findings of this study are available from the corresponding author upon reasonable request.
