# Supplementary material for: Piezoelectric Heterojunctions as Bacteria‐Killing Bone‐Regenerative Implants
Source: Adv Mater. 2024 Oct 25;37(1):2413171. doi: 10.1002/adma.202413171 (PMC11707579; doi:10.1002/adma.202413171)
Supplement: Supplementary file 1 — Supporting Information [file ADMA-37-2413171-s001.pdf]

ADVANCED MATERIALS

Supporting Information

for *Adv. Mater.*, DOI 10.1002/adma.202413171

Piezoelectric Heterojunctions as Bacteria-Killing Bone-Regenerative Implants

Youzhun Fan, Jinxia Zhai, Zhengao Wang, Zhaoyi Yin, Haoyan Chen, Maofei Ran, Zurong Zhu, Yubin Ma, Chengyun Ning, Peng Yu* and Chuanbin Mao**

Piezoelectric Heterojunctions as Bacteria-Killing Bone-Regenerative Implants

Youzhun Fan, Jinxia Zhai, Zhengao Wang, Zhaoyi Yin, Haoyan Chen, Maofei Ran, Zurong Zhu, Yubin Ma, Chengyun Ning, Peng Yu*, Chuanbin Mao**

Y. Fan, J. Zhai, Z. Wang, H. Chen, M. Ran, Z. Zhu, C. Ning, P. Yu

School of Materials Science and Engineering, GuangDong Engineering Technology Research Center of Metallic Materials Surface Functionalization, National Engineering Research Center for Tissue Restoration and Reconstruction, Medical Devices Research and Testing Center, South China University of Technology, Guangzhou 510641, P. R. China

E-mail: imcyning@scut.edu.cn (C. Ning); imyup@scut.edu.cn (P. Yu)

Y. Ma, C. Mao

Department of Biomedical Engineering, The Chinese University of Hong Kong, Sha Tin, Hong Kong SAR, P. R. China

E-mail: cmao@cuhk.edu.hk (C. Mao)

Z. Yin

Faculty of Materials Science and Engineering, Kunming University of Science and Technology, Kunming 650093, China

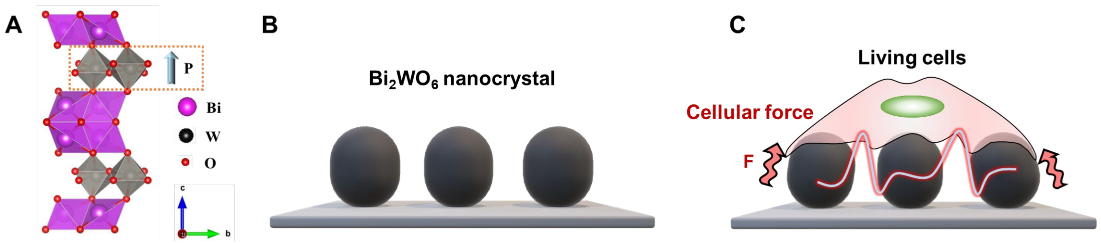

Figure S1. Construction and characterization of piezoelectric heterojunction model (TiO_2/Bi_2WO_6 model). A) The crystal structure of piezoelectric Bi_2WO_6. B) Schematic diagram of the heterojunction model, which is the Bi_2WO_6 piezoelectric nanocrystal on TiO_2 surface. C) Schematic diagram of living cells grown on the model heterojunction, showing the tilt of the nanocrystal by cellular force.

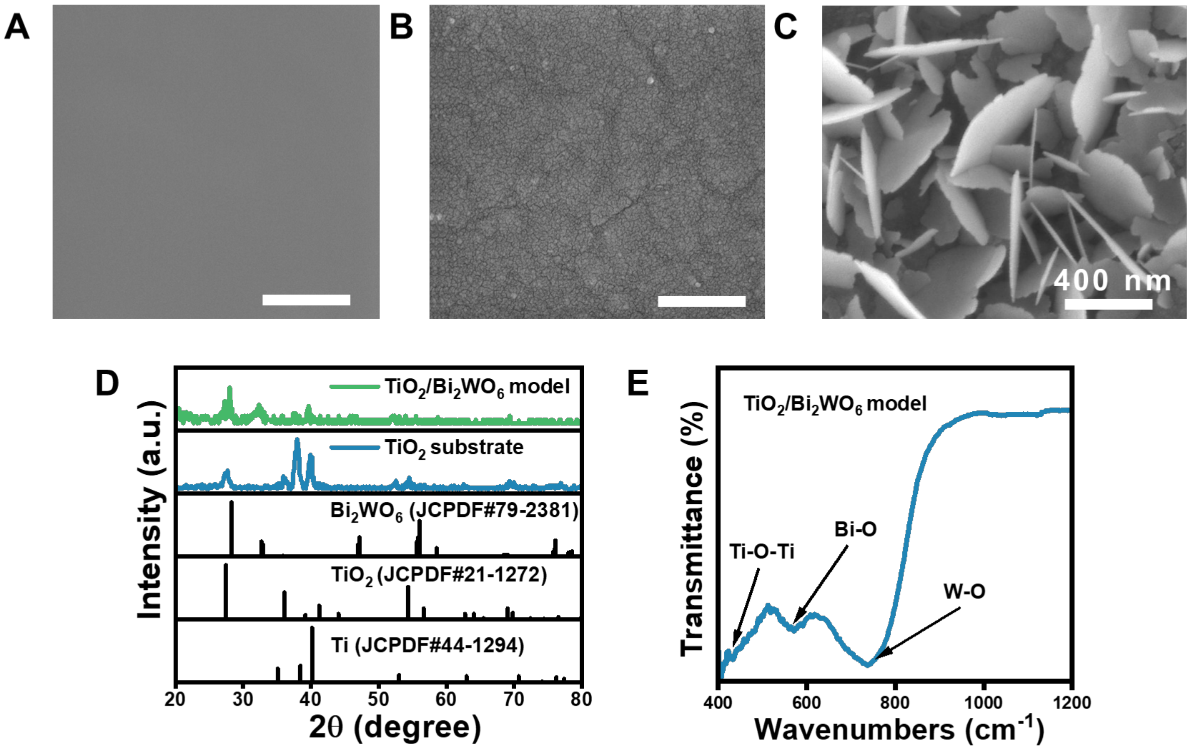

Figure S2. FE-SEM images of A) pure Ti, B) TiO_2 substrate, and C) TiO_2/Bi_2WO_6 model**.** D) XRD pattern of TiO_2 substrate and TiO_2/Bi_2WO_6 model**.** E) The FT-IR spectra of TiO_2/Bi_2WO_6 model.

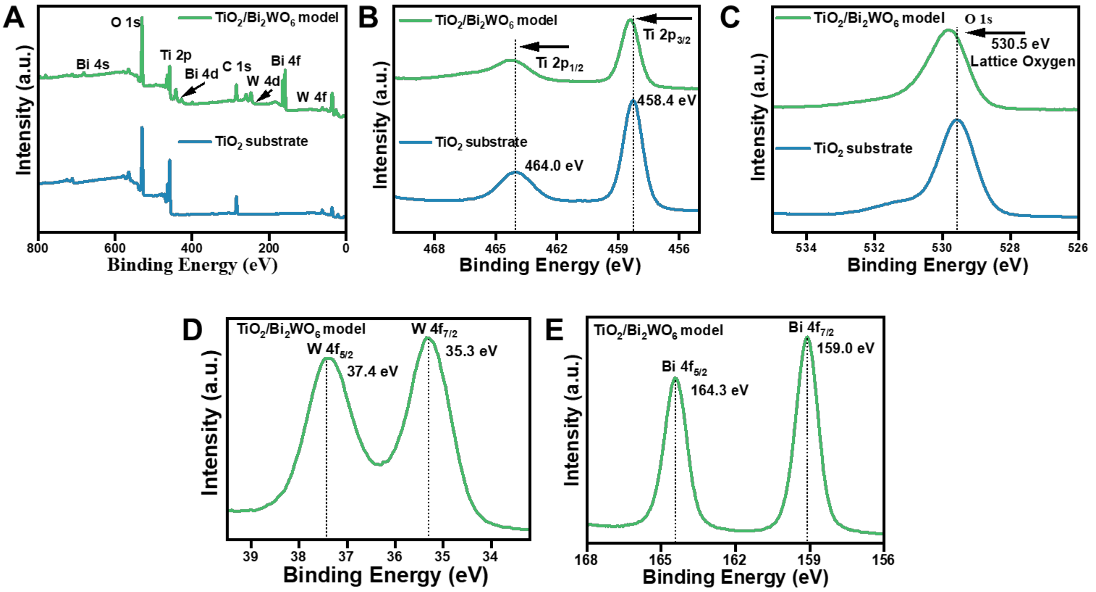

Figure S3. XPS spectra of TiO_2 substrate and TiO_2/Bi_2WO_6 model: A) survey spectra, B) Ti 2p, C) O 1s, D) W 4f, and E) Bi 4f.

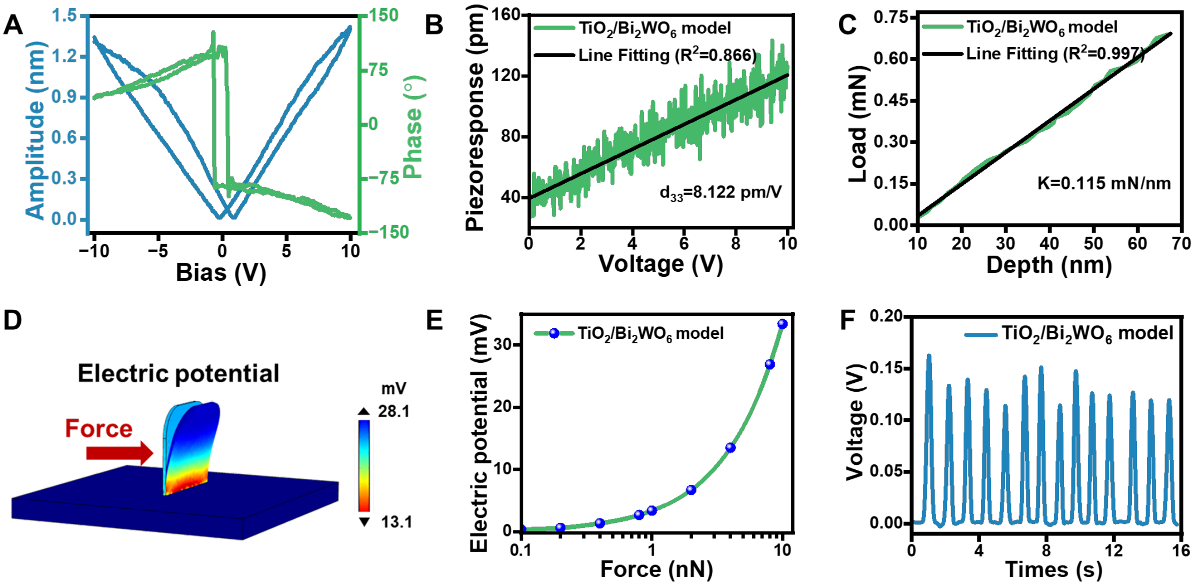

Figure S4. A) The local piezoelectric hysteresis loops, and B) the piezoelectric response versus applied voltage of the TiO_2/Bi_2WO_6 model. C) *In-situ* nanoindentation measurement on top of TiO_2/Bi_2WO_6 model. D) COMSOL simulation model of piezoelectric Bi_2WO_6 nanocrystal generating potential under simulated cellular force. E) Piezoelectric potential generated by TiO_2/Bi_2WO_6 model as a function of the applied cellular force. F) Mechanical–electric response properties of the TiO_2/Bi_2WO_6 model under force (8 Pa).

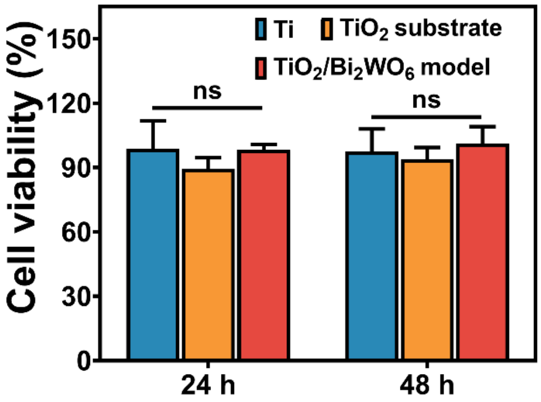

Figure S5. Cytotoxicity of TiO_2/Bi_2WO_6 model analyzed by CCK-8 assays of mBMSCs co-culture at 24 and 48 h.

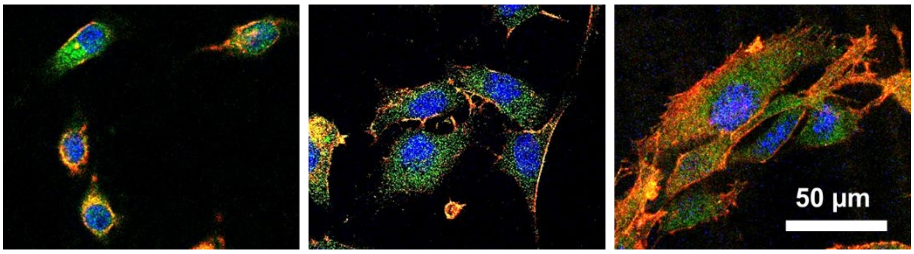

Figure S6. Immunofluorescence staining of vinculin (focal contacts, red) and F-actin (stress fibres, green) was detected after 2 days of co-culture with mBMSCs on Ti, TiO_2 substrate, and TiO_2/Bi_2WO_6 model using CLMS.

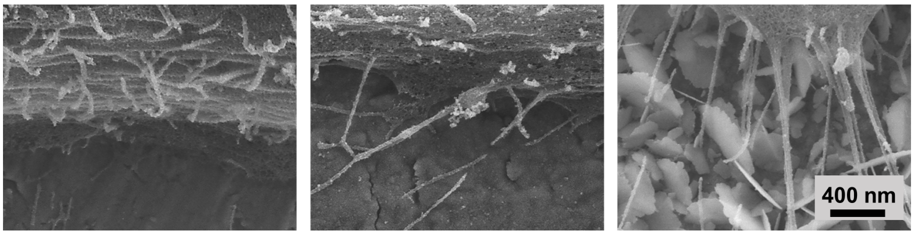

Figure S7. Adhesion morphology of mBMSCs with Ti, TiO_2 substrate, TiO_2/Bi_2WO_6 model after 2 days of co-culture by FE-SEM**.**

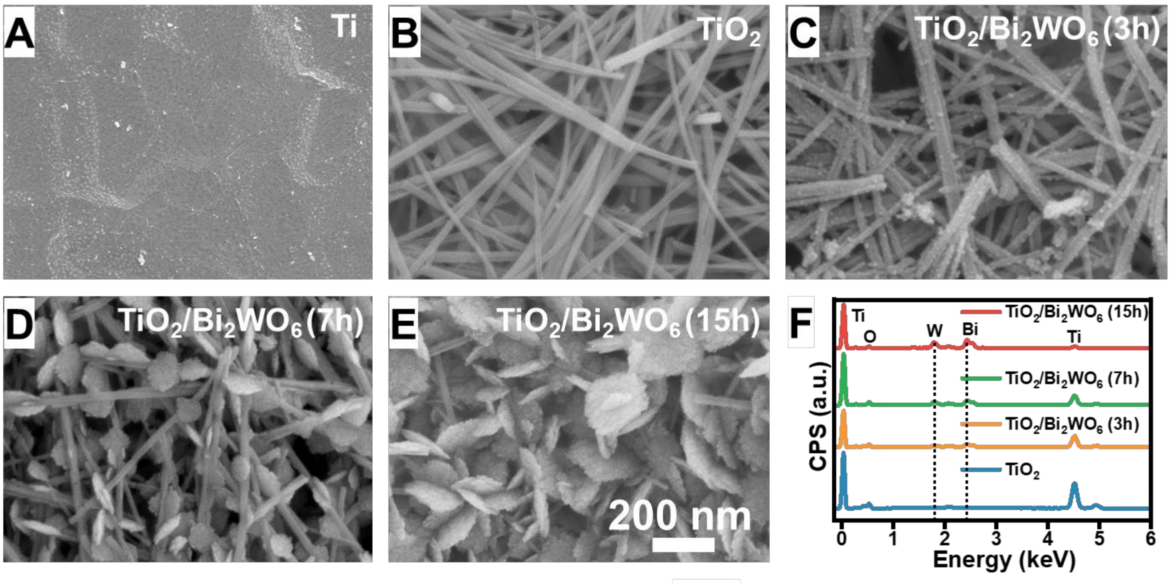

Figure S8. A, B, C, D, E) SEM images of all samples. F) EDX elemental mapping images of TiO_2, TiO_2/Bi_2WO_6 (3 h), TiO_2/Bi_2WO_6 (7 h), and TiO_2/Bi_2WO_6 (15 h).

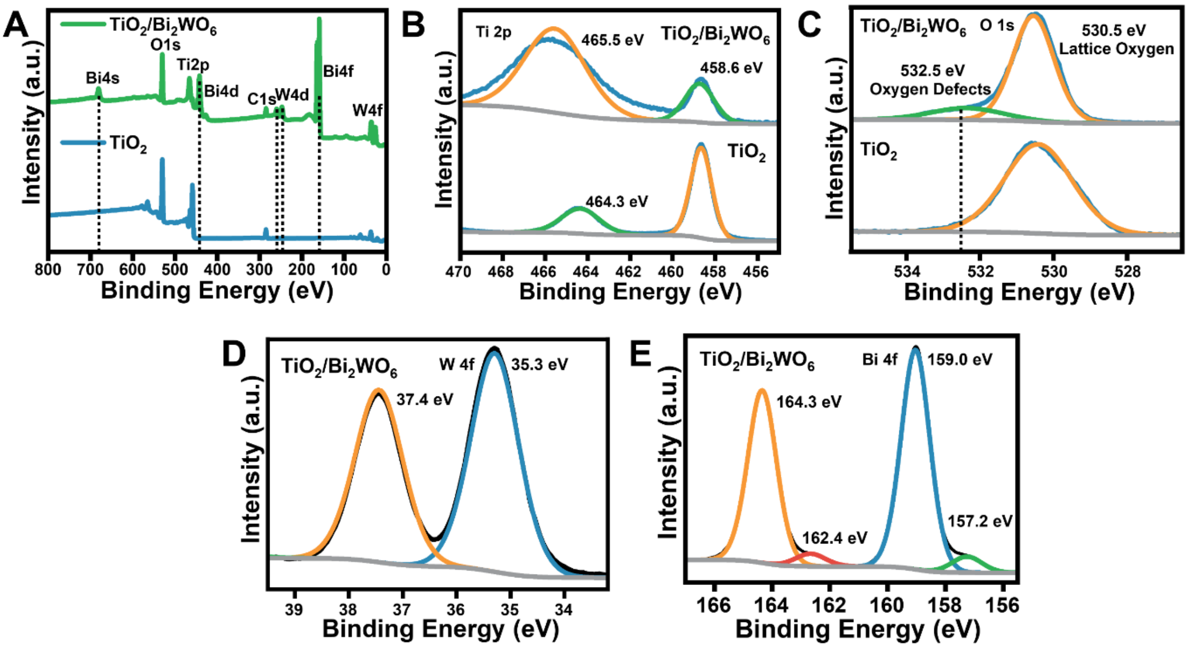

Figure S9. XPS spectra of TiO_2 and TiO_2/Bi_2WO_6: A) survey; B) Ti 2p; C) O 1s, D) W 4f and E) Bi 4f.

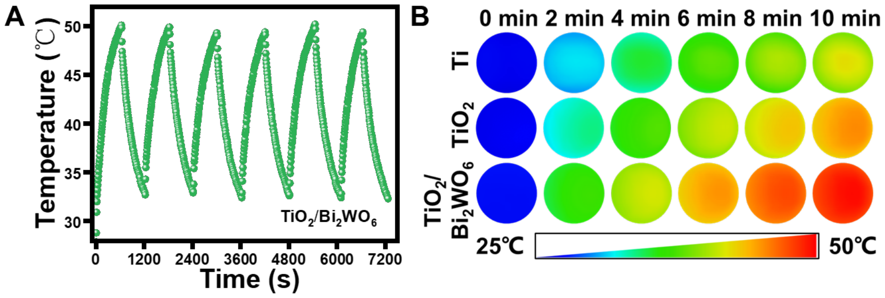

Figure S10. A) Repeated heating/cooling curves of TiO_2/Bi_2WO_6 heterojunctions. B) Real-time infrared thermal images.

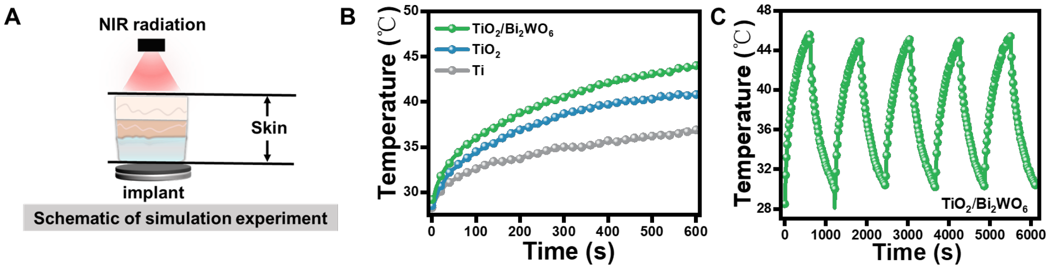

Figure S11. A) Schematic diagram of *in vivo* simulation experiment device. B) Photothermal curves of samples and C) repeated heating/cooling curves of TiO_2/Bi_2WO_6 heterojunctions under NIR irradiation *in vivo* simulation experiment device.

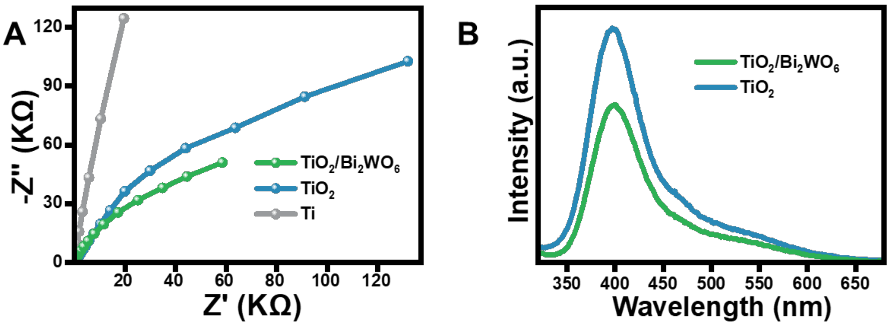

Figure S12. A) Nyquist diagram of electrochemical impedance. B) Photoluminescence spectra under excitation at 254 nm.

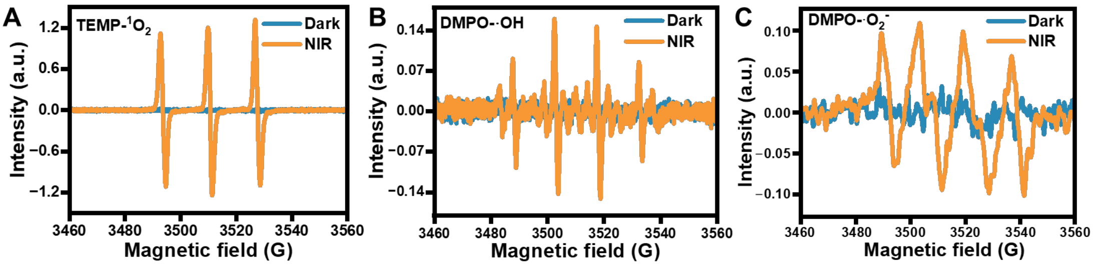

Figure S13. A) $^1O_2$, B) •OH, and C) $•O^{2-}$ from ESR spectra of TiO_2/Bi_2WO_6 heterojunctions under NIR irradiation.

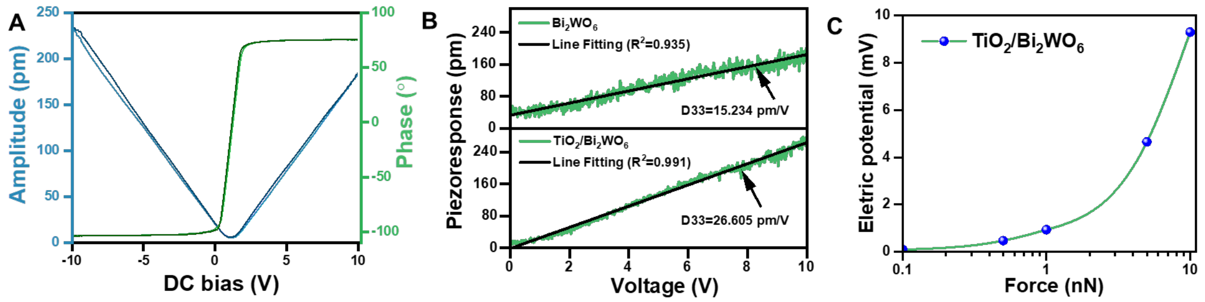

Figure S14. A) The local piezoelectric hysteresis loops of Bi_2WO_6. B) The piezoelectric response versus applied voltage of the Bi_2WO_6 and TiO_2/Bi_2WO_6. C) The piezoelectric potential generated by TiO_2/Bi_2WO_6 heterojunctions as a function of the applied force mimicking the cellular force traction.

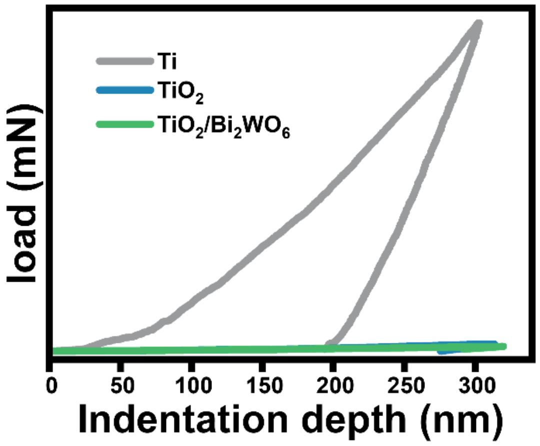

Figure S15. Young's modulus assay of Ti, TiO_2, and TiO_2/Bi_2WO_6 heterojunctions.

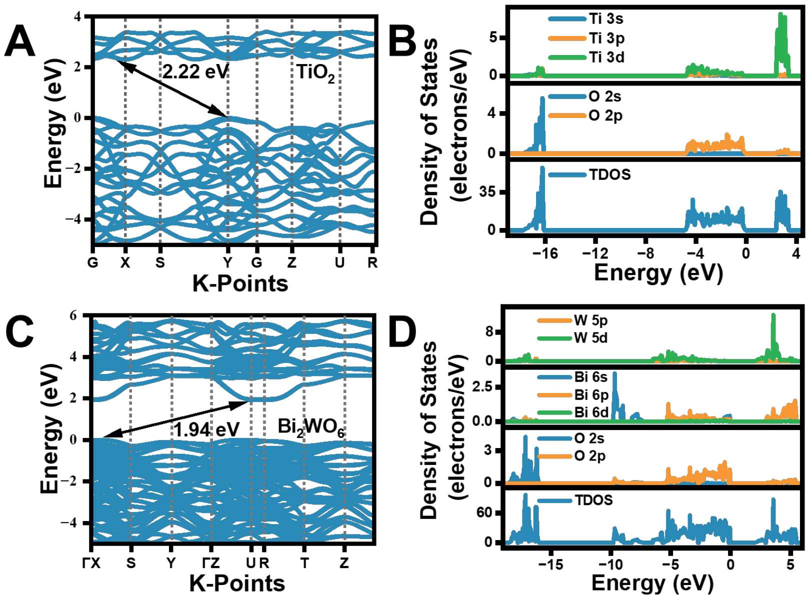

Figure S16. The band structure and the corresponding density of states (DOS) for orbital of A, B) TiO_2 and C, D) Bi_2WO_6.

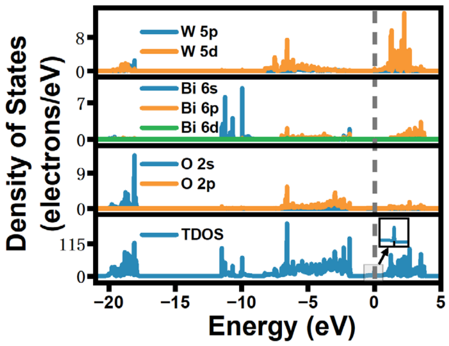

Figure S17. The density of states (DOS) of OVs-Bi_2WO_6.

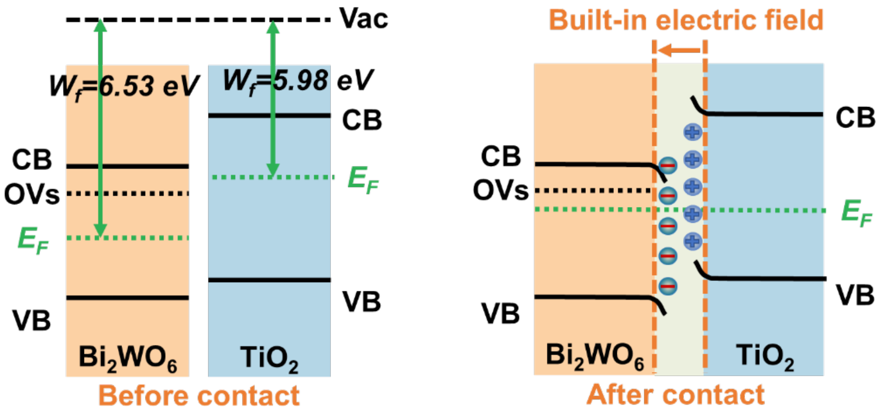

Figure S18. Schematic diagrams of charge transfer driven by potential difference and the formation of IEF of the TiO_2/Bi_2WO_6 heterojunction.

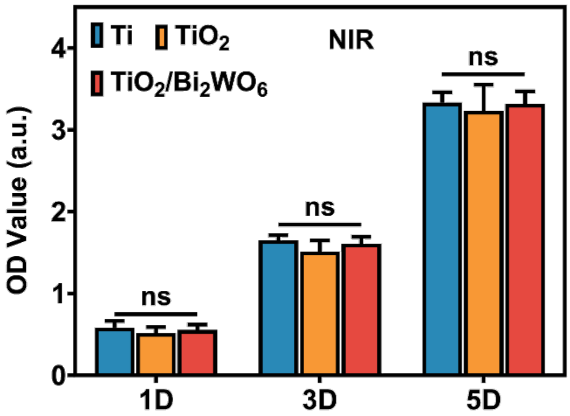

Figure S19. Cytotoxicity of all samples after co-culturing for 1, 3, and 5 days with mBMSC cells by CCK-8 assays under NIR irradiation.

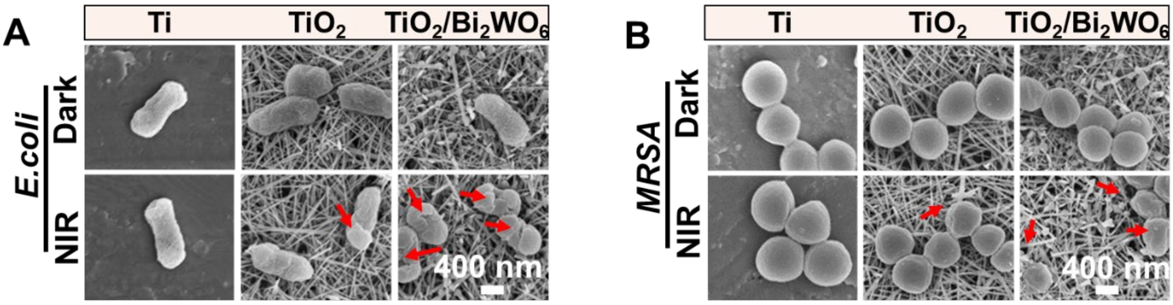

Figure S20. The SEM images of A) *E. coli* and B) *MRSA* on piezoelectric heterojunction surfaces. The red arrows represent the sites of damage to the bacteria.

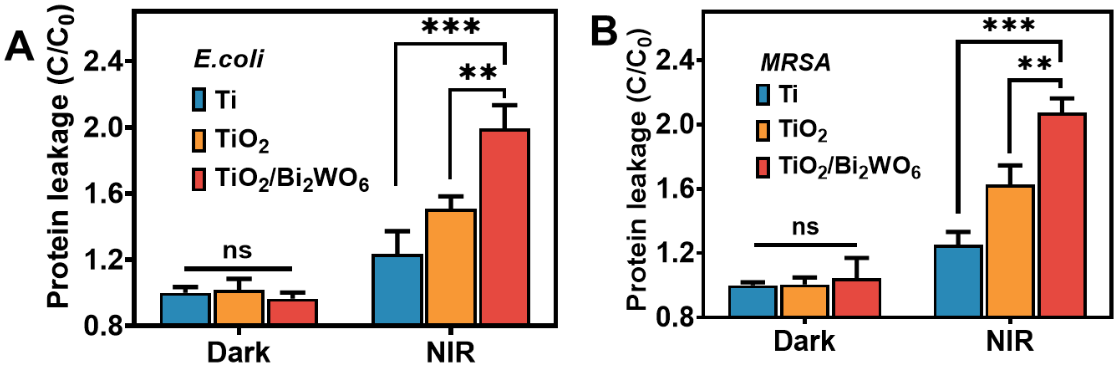

Figure S21. Protein leakage of C) *E. coli* and D) *MRSA* on piezoelectric heterojunctions. The values indicated means ± standard deviations (n = 3): ns represented p > 0.05, *p ≤ 0.05, **p < 0.01, and ***p < 0.001.

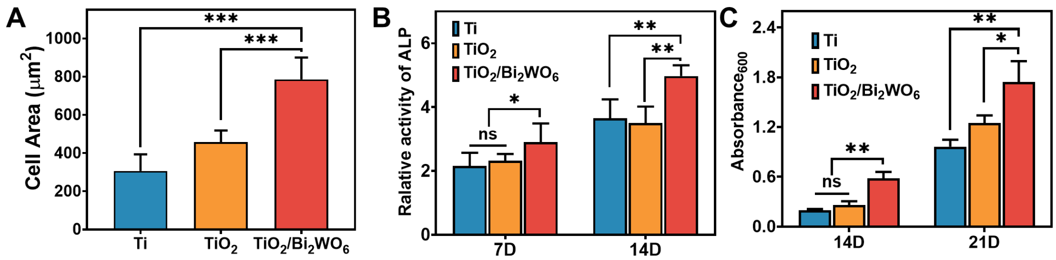

Figure S22. A) Cell spreading area was calculated by ImageJ software (n = 3). B) ALP activity assay of mBMSC cultured on the different sample surfaces for 7 and 14 days (n = 3). C) Calcium nodule activity assay of mBMSC cultured on the different sample surfaces for 14 and 21 days (n = 3). The values indicated means ± standard deviations (n = 3): ns represented *p* > 0.05, *$p \le 0.05$, **$p < 0.01$, and ***$p < 0.001$.

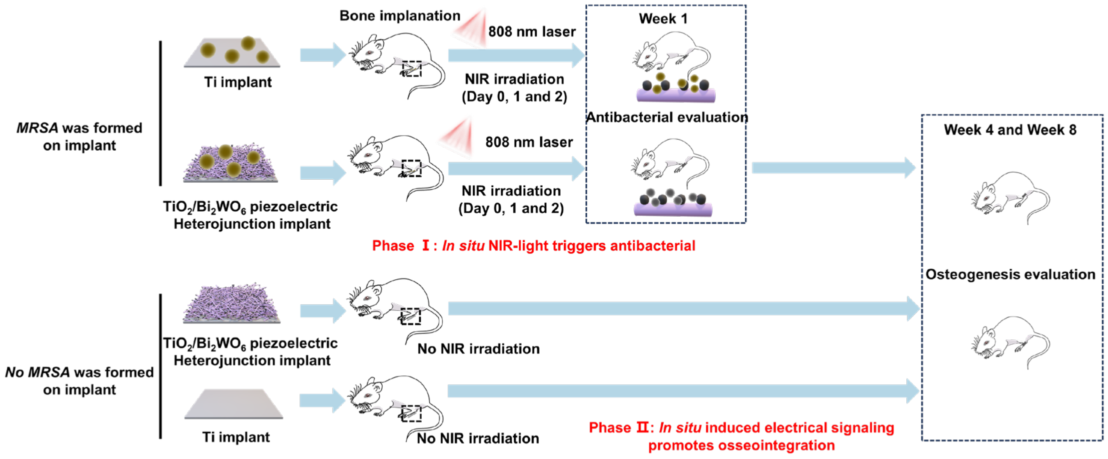

Figure S23. Schematic diagram of the animal experiment.

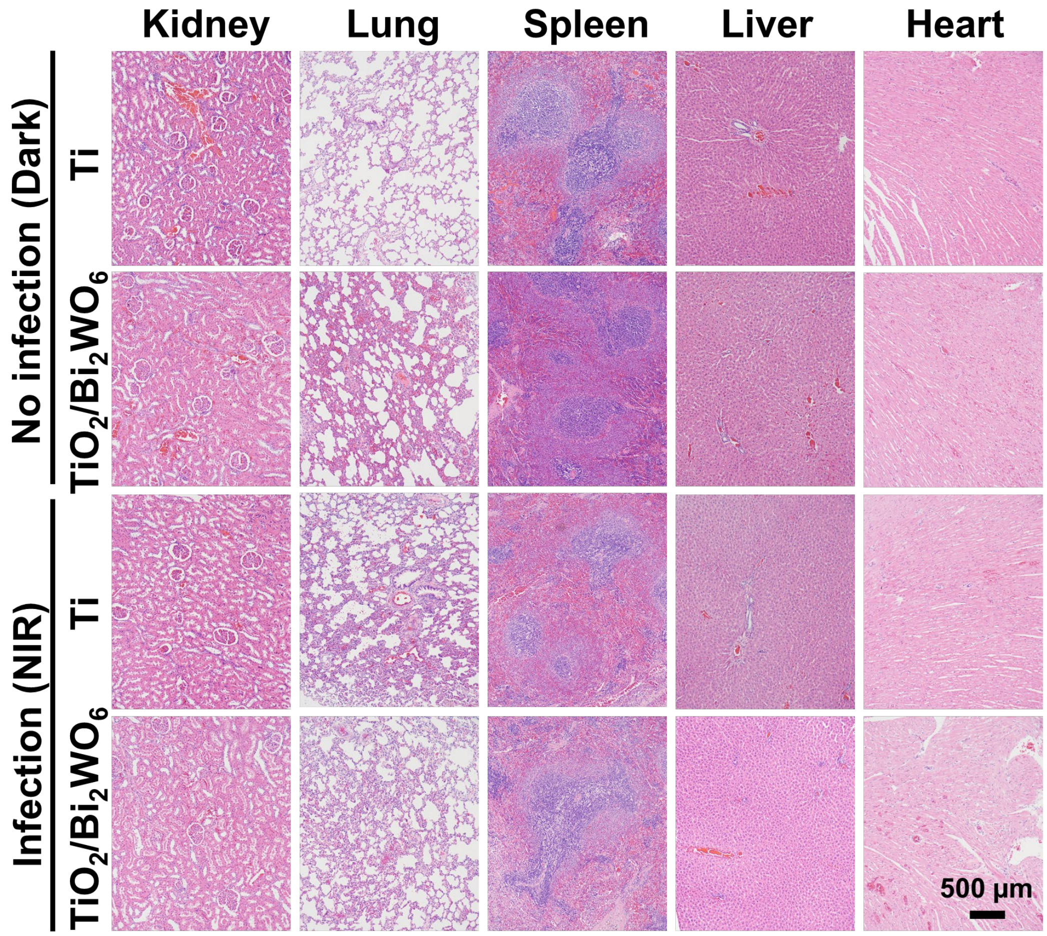

Figure S24. H&E staining of the heart, liver, spleen, lung and kidney tissues in different groups after 8 weeks of implantation.

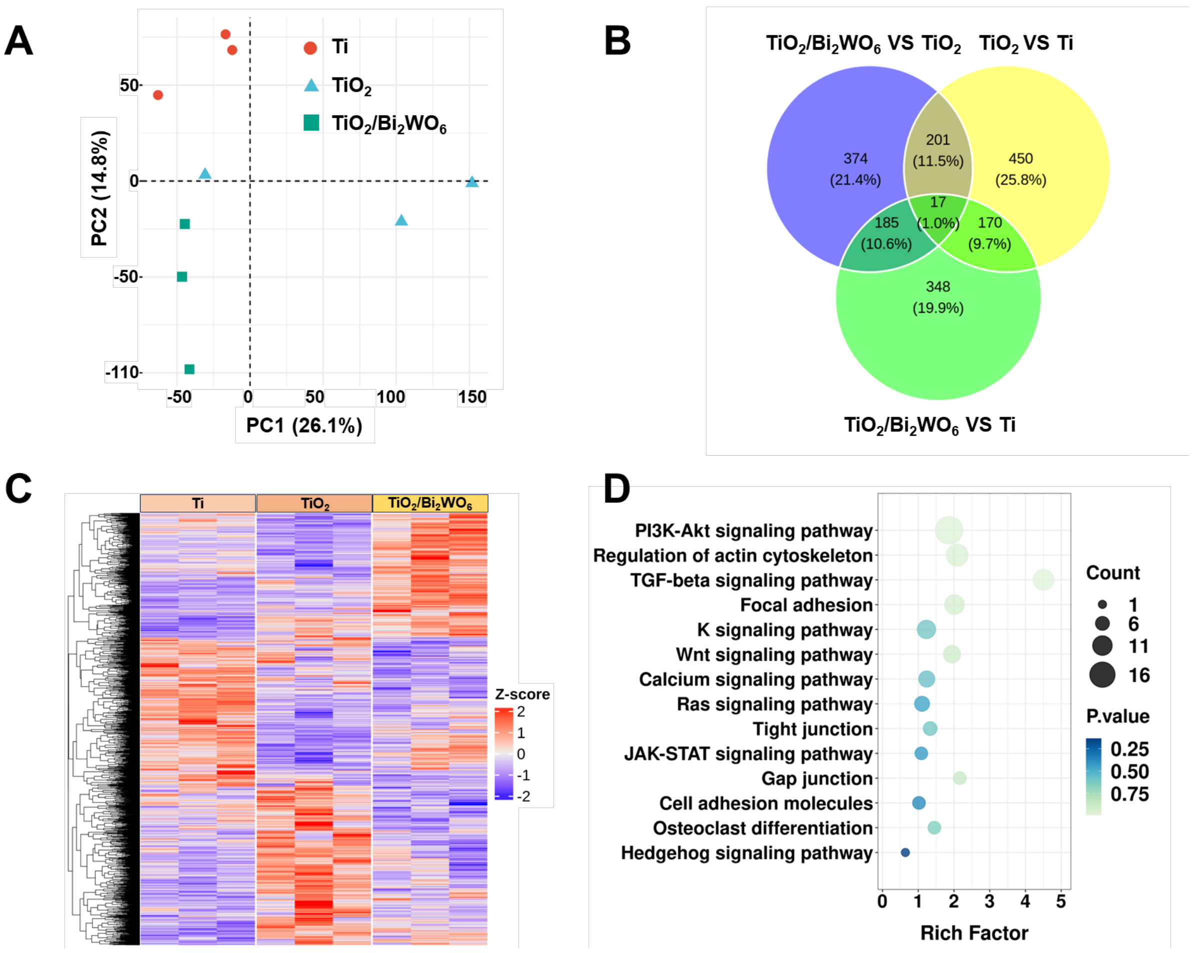

Figure S25. A) 2D principal component analysis (PCA) plot showing a correlation between samples. B) Venn diagram of the comparison of genes of the Ti, TiO_2, and TiO_2/Bi_2WO_6 groups. C) Heatmap of DEGs. D) The KEGG biological process enrichment analysis of the upregulated and downregulated DEGs of the TiO_2 versus Ti group.

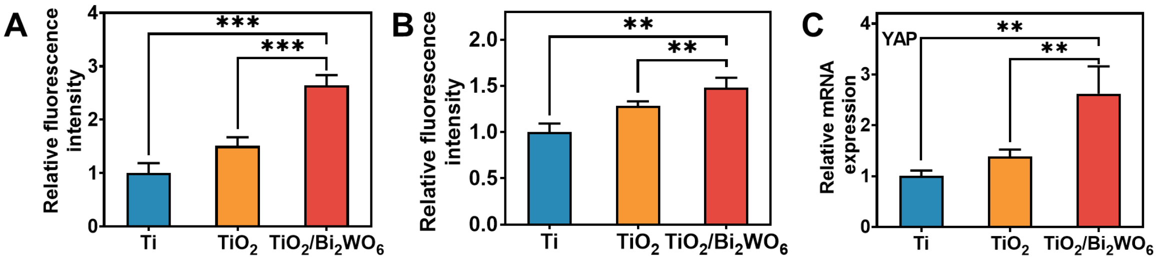

Figure S26. A) Intracellular Ca^{2+} staining quantitative analysis of the intracellular Ca^{2+} fluorescence intensity and B) cell membrane potential quantitative analysis cell membrane potential intensity of mBMSCs cells cultured on the different sample surfaces for 48 h. C) The YAP gene expression in mBMSCs cells on day 7. The data represented means ± standard deviations ($n = 3$) with ns indicating a p value of higher than 0.05. *$p \leq 0.05$, **$p < 0.01$, and ***$p < 0.001$.
